# Rhoifolin Alleviates Ulcerative Colitis by Targeting NMNAT1 and Activating SIRT1‐FOXO1 Signaling to Enhance ILC3s Effector Function

**DOI:** 10.1002/advs.76935

**Published:** 2026-08-07

**Authors:** Hongqiong Yang, Yishu Zhang, Sijia Feng, Xiaoqing Ma, Jiayi Feng, Junwei Wang, Jialin Gao, An Pan, Dapeng Wu, Kang Ding, Caijuan Zheng, Qi Lv, Lihong Hu

**Affiliations:** ^1^ Jiangsu Key Laboratory For Functional Substance of Chinese Medicine School of Pharmacy Nanjing University of Chinese Medicine Nanjing People's Republic of China; ^2^ Jiangsu Province Hospital of Chinese Medicine Affiliated Hospital of Nanjing University of Chinese Medicine Nanjing People's Republic of China; ^3^ Nanjing Hospital of Chinese Medicine Affiliated to Nanjing University of Chinese Medicine Nanjing People's Republic of China; ^4^ College of Chemistry and Chemical Engineering Key Laboratory of Tropical Medicinal Plant Chemistry of Ministry of Education Hainan Normal University Haikou People's Republic of China

**Keywords:** IL‐22, ILC3s, NMNAT1, rhoifolin, SIRT1‐FOXO1 axis, ulcerative colitis

## Abstract

Ulcerative colitis (UC) is a chronic inflammatory bowel disorder with limited therapeutic options, particularly those capable of selectively reinforcing intestinal epithelial barrier integrity while preserving immune homeostasis. Here, Rhoifolin (3, 10 mg/kg) dose‐dependently alleviates DSS and TNBS‐induced colitis in mice. Mechanistically, Rhoifolin selectively potentiates ILC3s effector function without affecting their proliferation, apoptosis, intestinal homing, developmental differentiation, or the distribution of CCR6^+^, NKp46^+^, and double‐negative ILC3s subsets. This functional enhancement promotes IL‐22 production, thereby reinforcing epithelial barrier integrity in colonic epithelial cells and organoids. Integrated transcriptomics and untargeted metabolomics identified nicotinamide salvage as a central metabolic program driving ILC3s functional enhancement. NMNAT1 was identified as a direct molecular target of Rhoifolin. Molecular docking and dynamic simulations pinpointed Leu155 as a critical residue mediating Rhoifolin‐NMNAT1 interaction, and mutation of Leu155 antagonized metabolic activation and IL‐22 induction. Consistently, Rhoifolin‐induced NAD+ elevation enhanced SIRT1 activity, leading to FOXO1 deacetylation and nuclear translocation. Further CUT&Tag‐qPCR demonstrated FOXO1 enrichment at *Il22* promoter, driving IL‐22 transcription in ILC3s. Collectively, our findings identify Rhoifolin as a potent ILC3s effector function enhancer and uncover a previously unrecognized NMNAT1‐NAD+‐SIRT1‐FOXO1 axis linking immunometabolic and epigenetic regulation to intestinal barrier protection, highlighting ILC3s empowerment as a promising therapeutic strategy for UC.

## Graphical abstract

Rhoifolin directly targets NMNAT1, thereby activating nicotinamide salvage pathway and promoting intracellular NAD+ biosynthesis. Elevated NAD^+^ levels enhance SIRT1 activity, leading to FOXO1 deacetylation and nuclear translocation. Nuclear FOXO1 subsequently binds to the IL‐22 promoter, upregulating IL‐22 transcription and potentiating ILC3 effector function, which in turn restores intestinal epithelial barrier integrity and ultimately alleviates UC.

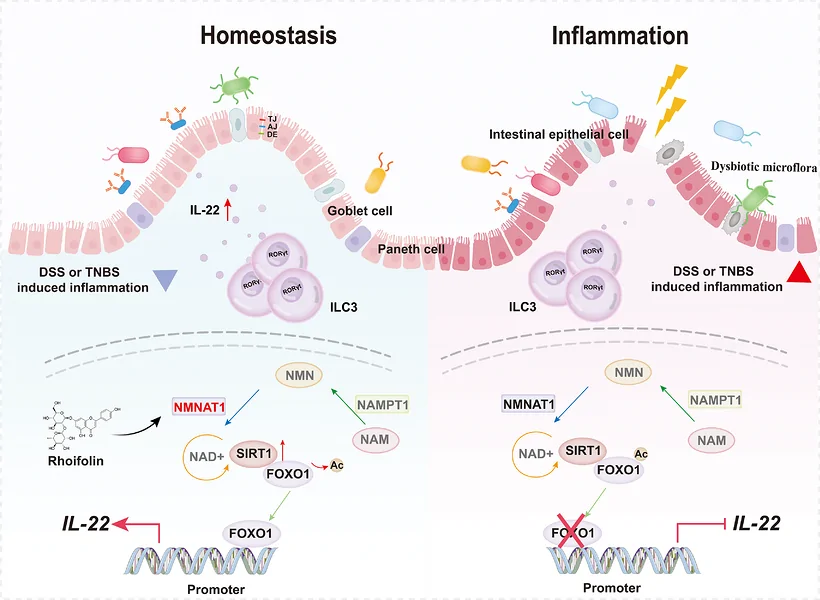

## 1 Introduction

Ulcerative colitis (UC) is a chronic, relapsing inflammatory bowel disease (IBD) characterized by continuous mucosal inflammation and ulceration of the colon. Clinically, UC commonly presents with abdominal pain, diarrhea, rectal bleeding, and body weight loss. Epidemiological studies have documented a steadily rising incidence and prevalence of UC worldwide, particularly in industrialized countries, posing a growing burden on public health systems. Pathologically, UC is defined by epithelial barrier integrity disruption, excessive immune cell infiltration, and uncontrolled release of pro‐inflammatory cytokines, contributing to sustained mucosal damage and chronic inflammation [1]. Despite substantial advances in understanding the pathogenesis of UC, current treatments, including corticosteroids, immunosuppressants, and biologics remain limited by insufficient efficacy, frequent relapse, and adverse effects. Therefore, novel therapeutic strategies with improved efficacy and safety are urgently needed.

Group 3 innate lymphoid cells (ILC3s) are specialized innate lymphoid cells that play indispensable roles in mucosal immunity and intestinal homeostasis [2]. Derived from common lymphoid progenitors, ILC3s are defined by the transcription factor RORγt and produce multiple effector cytokines, particularly IL‐22 and IL‐17, in response to inflammatory and microbial cues [3]. Among these cytokines, IL‐22 is particularly important for epithelial barrier maintenance and repair in the intestine. IL‐22 primarily acts on epithelial cells and exerts barrier‐protective effects by promoting epithelial proliferation [4], goblet cell differentiation [5], mucin production [6], and antimicrobial peptide expression, including RegIIIγ [7, 8], thereby reinforcing barrier integrity and limiting microbial translocation. Consistently, exogenous administration or enhanced expression of IL‐22 ameliorates colitis severity in mice [9, 10, 11], whereas IL‐22 deficiency aggravates epithelial injury and mucosal inflammation [12]. In parallel, IL‐17A has been implicated in mucosal immune activation and inflammatory responses [13, 14]. Thus, modulating ILC3s effector function, particularly enhancing IL‐22‐associated barrier repair, may represent a promising strategy for restoring epithelial homeostasis in UC. However, the intrinsic mechanisms that regulate IL‐22 production by ILC3s during intestinal inflammation remain incompletely understood.

*Callicarpa nudiflora*, a traditional medicinal herb native to Hainan Province, has long been valued in Chinese medicine for its unique pharmacological properties. Among its bioactive constituents, Rhoifolin has attracted increasing attention due to its anti‐inflammatory, antioxidant, and hepatoprotective effects [15]. However, its therapeutic potential in colitis and the underlying mechanisms remain largely unexplored. In the present study, we demonstrated for the first time that Rhoifolin markedly alleviated colitis induced by dextran sulfate sodium (DSS) and 2, 4, 6‐trinitrobenzene sulfonic acid (TNBS) in mice, with pronounced restoration of the intestinal epithelial barrier integrity. Further in vitro experiments revealed that Rhoifolin alone exerted no significant effect on TNF‐α‐induced epithelial barrier disruption; however, conditioned medium from co‐cultures with ILC3s markedly mitigated epithelial injury. These findings suggest that Rhoifolin protects the intestinal barrier indirectly by potentiating ILC3s effector function, particularly IL‐22 production. Given the limited understanding of the molecular mechanisms governing ILC3s‐derived IL‐22 production, we used Rhoifolin as a chemical probe to dissect the intrinsic regulatory pathways that control ILC3s‐mediated epithelial repair in UC. This probe‐based approach reveals a previously unrecognized metabolic‐transcriptional axis underlying ILC3s‐dependent mucosal protection and highlights the therapeutic potential of targeting ILC3s metabolic programs for UC treatment.

## 2 Materials and Methods

### 2.1 Animals

Female C57BL/6 mice (6–8 weeks old, weighing 18–22 g) were purchased from Shanghai Laboratory Animal Center (Shanghai, China). The animals were housed under standard laboratory conditions with a 12‐h light/dark cycle, controlled temperature (20–24°C), and relative humidity (40–60%). Mice were provided ad libitum access to standard chow and water and were acclimatized to the housing environment for one week prior to experimentation. All procedures were conducted in strict compliance with the guidelines of the Ethics Committee of Nanjing University of Chinese Medicine and approved under protocol numbers 202503A062 and 202504A049. All efforts were made to minimize animal discomfort and to reduce the number of animals used.

### 2.2 Establishment of Colitis Model and Treatment Protocol

Acute colitis was induced in female C57BL/6 mice by administering 2.5% (w/v) dextran sulfate sodium (DSS, molecular weight: 36,000–50,000 Da, MP Biomedicals, Santa Ana, CA, USA) in drinking water for 7 consecutive days. For the chronic colitis model, female C57BL/6 mice were subjected to three cycles of DSS administration. Each cycle consisted of 2.0% (w/v) DSS in drinking water for 5 consecutive days, followed by regular drinking water for 10 days to allow partial recovery. Fresh DSS solution was prepared every two days to ensure consistent exposure. (1) To determine the protective effect of Rhoifolin on colitis, the mice were randomly divided into six groups: Normal, DSS, Rhoifolin (1, 3, 10 mg/kg) and 5‐ASA (200 mg/kg) groups. Rhoifolin (17306‐46‐6, purity >98%) was purchased from Chengdu Push Bio‐Technology (PU0702‐0025, Chengdu, China) and suspended in 0.5% CMC‐Na. Mice were orally administered Rhoifolin once daily from day 1 of DSS administration until the end of experiment. Mice in Normal and DSS‐only groups received an equivalent volume of vehicle for consistency; (2) To assess the role of IL‐22 in protective effects of Rhoifolin against UC, mice were randomly divided into five groups: Normal, DSS, IL‐22 neutralizing antibody, IL‐22 neutralizing antibody + Rhoifolin, and Rhoifolin (10 mg/kg). For IL‐22 blockade, mice received intraperitoneal injection of 20 µg anti‐IL‐22 monoclonal antibody (clone IL22JOP, BioXcell, West Lebanon, NH, USA) per mouse. Antibody administration was performed five times in total: one day before DSS induction (day ‐1), on the first day of DSS induction (day 0), and subsequently on days 3, 6, and 9. Control animals received isotype‐matched IgG on the same schedule; (3) To evaluate the role of CD4^+^ T cells in protective effects of Rhoifolin against UC, mice were randomly assigned to five groups: Normal, DSS, anti‐CD4^+^ T, Rhoifolin (10 mg/kg), and anti‐CD4^+^ T + Rhoifolin. CD4^+^ T cells were depleted by intraperitoneal injection of 100 µg anti‐CD4 monoclonal antibody (clone GK1.5, BioXcell, West Lebanon, NH, USA) per mouse. Antibody administration was performed three times in total: once 3 days before DSS induction (day ‐3), again on the first day of DSS administration (day 0), and a final injection on day 3 after DSS induction. Control mice received isotype‐matched IgG under the same schedule. 4) To investigate the functional importance of NMNAT1 in protective effects of Rhoifolin against UC, mice were randomly assigned to five groups: Normal, DSS, AAV9‐*Nmnat1* shRNA, AAV9‐*Nmnat1* shRNA + Rhoifolin, and Rhoifolin (10 mg/kg). For NMNAT1 knockdown, an AAV9 vector carrying the NMNAT1‐targeting shRNA was administered via rectal injection 30 days prior to DSS induction (single dose). Control mice received an AAV9‐scramble shRNA following the same procedure.

Body weight, stool consistency, and the presence of blood in feces were monitored daily to calculate the disease activity (DAI) scores. All assessments were performed by investigators blinded to the group assignments to minimize observer bias. Scoring criteria were standardized and applied consistently across all animals.

### 2.3 Adoptive Transfer of ILC3s in Colitis Mice

ILC3s were purified from intestinal lamina propria of wild‐type donor mice and maintained in RPMI 1640 medium supplemented with 10% FBS, 1% p/s, recombinant murine IL‐7 (20 ng/mL), IL‐2 (10 ng/mL), and IL‐15 (10 ng/mL) to support cell survival and proliferation. The purified cells were exposed to either Rhoifolin (30 µM) or vehicle control for 48 h prior to adoptive transfer. For adoptive transfer, the recipient mice received intravenous injections of 1×10^6^ Rhoifolin‐pretreated or untreated ILC3s via the tail vein on days ‐2, 2, 5 relative to DSS initiation. Disease progression was monitored daily by assessing body weight, stool consistency, and rectal bleeding to calculate DAI scores. On day 10, mice were sacrificed and colons were harvested for histopathology and immunofluorescence analysis.

### 2.4 Intestinal Permeability Assay Using FITC‐dextran

At the end of experiment, mice were fasted for 6 h with free access to water. FITC‐dextran, 4 kDa (FD4, 60842‐46‐8, Sigma–Aldrich, St. Louis, MO, USA) was dissolved in sterile PBS and administered orally at a dosage of 50 mg/kg. 4 h post‐gavage, blood samples were collected from the retroorbital sinus under isoflurane anesthesia and transferred to heparin‐coated tubes. The plasma was separated by centrifugation at 3000 rpm for 20 min, and fluorescence intensity was measured using a microplate reader with excitation wavelength at 485 nm and emission wavelength at 535 nm. Plasma FD4 concentrations were determined based on a standard curve generated under identical conditions.

### 2.5 Colonoscopy

Colonic inflammation was evaluated using a small animal colonoscopy system. Mice were anesthetized with isoflurane to ensure immobilization and minimize procedural stress. After cleaning the anal area, a lubricated flexible endoscope was gently inserted into the rectum and advanced proximally along the colon. Real‐time imaging enabled evaluation of mucosal pathology, including erythema, granularity, ulceration and bleeding.

### 2.6 Transmission Electron Microscopy

To investigate the ultrastructural integrity of the colonic epithelial barrier in the murine colitis model, transmission electron microscopy (TEM) was utilized. Colonic tissues were harvested immediately after euthanasia and gently rinsed with PBS to remove residual fecal contents. Small tissue fragments were fixed overnight at 4°C in 2.5% glutaraldehyde to preserve fine cellular structures. Following fixation, samples were rinsed with PBS and post‐fixed in 1% osmium tetroxide for 2 h at room temperature to enhance membrane preservation. The specimens were then dehydrated through graded ethanol series and embedded in epoxy resin. Ultrathin sections (70 nm) were cut using an ultramicrotome, mounted on copper grids, and stained with uranyl acetate and lead citrate. The sections were examined under a TEM to evaluate epithelial ultrastructure, including tight junction morphology and microvilli organization.

### 2.7 Bacterial Culture

At the end of experiment, spleens and mesenteric lymph nodes (MLNs) were aseptically harvested from colitis mice. The tissues were mechanically homogenized under sterile conditions at 4°C for 1 min. The resulting homogenate was serially diluted in sterile PBS and plated onto Luria–Bertani (LB) agar plates, followed by incubation at 37°C for 12 h. Bacterial colonies in each group were counted to determine microbial load.

### 2.8 Fluorescence In Situ Hybridization (FISH)

Freshly harvested colonic tissues were carefully rinsed with ice‐cold PBS to remove luminal contents and fixed in 4% paraformaldehyde at 4°C for 24 h. Fixed tissues were embedded in paraffin and sectioned at 5 µm thickness. Sections were deparaffinized in xylene, rehydrated through a graded ethanol series (100%, 95%, 70%), and rinsed in distilled water. For permeabilization, sections were treated with 0.1% Triton X‐100 in PBS for 10 min, followed by incubation with 20 µg/mL proteinase K at 37°C for 15 min. Hybridization was performed in a humidified chamber at 46°C for 2 h using a buffer containing 20 mM Tris‐HCl (pH 7.4), 0.9 M NaCl, 0.1% SDS, and 30% formamide with 10 ng/µL EUB338 probe. After hybridization, slides were washed in pre‐warmed washing buffer (20 mM Tris‐HCl, 0.9 M NaCl, 0.1% SDS) at 48°C for 15 min to remove unbound probes. Sections were mounted with ProLong Gold Antifade Mountant containing DAPI for nuclear counterstaining, and confocal fluorescence microscopy was used to visualize bacterial localization.

### 2.9 Small Animal Imaging

To evaluate the effects of Rhoifolin on intestinal epithelial barrier integrity, fluorescence‐based small animal imaging was conducted. The mice were fasted for 6 h, and orally administered FITC‐dextran at a dose of 50 mg/kg to evaluate epithelial permeability. After 4 h, mice were anesthetized with isoflurane in an oxygen‐enriched chamber, and the systemic fluorescence reflecting tracer leakage into the circulation due to barrier dysfunction was measured using an IVIS Spectrum imaging system (PerkinElmer, Waltham, MA, USA). Following imaging, the mice were monitored until full recovery from anesthesia.

### 2.10 Isolation and Sorting of ILC3s

Single‐cell suspensions were prepared from intestinal lamina propria or spleens using enzymatic digestion. For the ILC3s gut homing experiments, these cells were isolated from spleens. For all experiments investigating ILC3s effector function, including IL‐22 expression and cytokine secretion, these cells were isolated from the intestinal lamina propria. The colons were opened longitudinally and thoroughly washed with ice‐cold PBS to remove fecal contents. Tissue segments (∼2 cm) were sequentially incubated in PBS containing 1 mM DTT and PBS containing 30 mM EDTA at 37 °C with shaking at 220 rpm for 10 min each, followed by vigorous manual shaking to remove mucus and epithelial cells. The tissues were further minced into smaller fragments and digested in RPMI 1640 medium supplemented with 200 U/mL collagenase VIII and 150 µg/mL DNase I at 37 °C for 90 min without agitation. The resulting cell suspension was filtered through a 70 µm cell strainer and centrifuged at 400 × g for 10 min at 4 °C. Mononuclear cells were enriched using a Percoll density gradient (40%/80%, 800 × g, 20 min, room temperature), and cells at the interphase were collected, washed with PBS, and resuspended in RPMI 1640 medium containing 10% FBS for further use; for the obtain of single‐cell suspensions from spleens, mice were euthanized, and spleens were gently dissociated by mechanical trituration through a 70 µm nylon cell strainer using the plunger of a 1 mL syringe, followed by rinsing with 5 mL PBS solution. Cell suspensions were pelleted and red blood cells were lysed with ACK lysis buffer (C3702, Beyotime, Shanghai, China) for 5 min at room temperature. The lysis was quenched with 10× volume PBS and cells were washed. The final pellet was resuspended in RPMI 1640 medium containing 10% FBS for further use.

For ILC3s sorting, cells were incubated with fluorochrome‐conjugated antibodies against CD3e, Ly6G/Ly6c, CD11b, CD11c, NK1.1, KLRG1, CD127, CD90.2, CD45 for 30 min at 4°C. Sorting was performed using the FACS Aria TM Fusion (BD Bioscience, Franklin Lake, NZ, USA). ILC3s were identified as Lin^−^ NK1.1^−^ KLRG1^−^ CD45^+^ CD127^+^ CD90.2^+^ cells, collected and placed in RPMI‐1640 medium supplemented with 10% FBS, and supplemented with recombinant murine IL‐7 (20 ng/mL), IL‐2 (10 ng/mL), and IL‐15 (10 ng/mL) to support cell survival and proliferation for subsequent in vitro culture. Purity of the sorted populations was confirmed by reanalyzing a fraction of the cells via flow cytometer, achieving >95% purity.

### 2.11 Evaluation of ILC3s Effector Function

To determine whether Rhoifolin enhances ILC3s effector function, the purified ILC3s were cultured in RPMI‐1640 medium supplemented with 10% FBS in the presence or absence of Rhoifolin for 48 h. After treatment, the culture supernatants were collected for IL‐22 quantification using a mouse IL‐22 ELISA kit (88‐7422‐88, Thermo Fisher Scientific, Waltham, MA, USA) according to the manufacturer's instructions. Briefly, 96‐well ELISA plates were pre‐coated with the capture antibody specific for IL‐22 and incubated overnight at 4°C. The next day, the plates were washed three times with washing buffer (0.05% Tween‐20 in PBS) and blocked with 200 µL of blocking buffer (1% BSA in PBS) for 1 h at room temperature. Following blocking, 100 µL of cell culture supernatants or IL‐22 standards were added to the wells and incubated for 2 h at room temperature. After incubation, the plates were washed five times with washing buffer, and 100 µL of biotin‐labeled detection antibody was added to each well. The plates were incubated for 1 h at room temperature, followed by five additional washes. Next, 100 µL of streptavidin‐horseradish peroxidase (HRP) conjugate was added to each well and incubated for 30 min at room temperature in the dark. After washing the plates seven times, 100 µL of the tetramethylbenzidine (TMB) substrate solution was added to each well and incubated for 10–15 min at room temperature to allow color development. The reaction was stopped by adding 50 µL of stop solution (2 M H_2_SO_4_), and the absorbance was measured at 450 nm using a microplate reader. IL‐22 concentrations in the samples were calculated by generating a standard curve using known concentrations of IL‐22.

### 2.12 Isolation and Culture of Colonic Epithelial Organoids

Colonic epithelial organoids were isolated and cultured according to established protocols with minor modifications. Briefly, colonic tissues were excised from adult mice and extensively rinsed with ice‐cold DPBS to remove luminal contents. The intestines were then opened longitudinally, and the luminal surface was gently scraped to eliminate residual mucus and superficial epithelial layers, followed by additional washing with DPBS. The cleaned tissues were subsequently cut into approximately 5‐mm fragments and incubated in DPBS containing 5 mM EDTA at 4°C for 30 min to promote epithelial crypt dissociation. After incubation, the tissue fragments were transferred to fresh tubes containing DPBS and washed thoroughly to remove residual EDTA. Crypts were released by gentle pipetting, and the resulting suspension was passed through a 70‐µm cell strainer to remove debris. Crypts were then collected by centrifugation at 300 × g for 5 min.

The isolated crypts were resuspended in growth factor‐reduced Matrigel and seeded (20–50 µL per well) onto pre‐warmed 24‐well plates. Following polymerization of the Matrigel at 37°C, 500 µL of pre‐warmed organoid culture medium was added to each well. The organoid culture medium was prepared using the Mouse Colonic Organoid Culture Kit (Biogenous). Specifically, 200 µL of Organoid Supplement B (50×), 40 µL of Supplement C (250×), and 40 µL of Supplement D (250×) were added to 9.72 mL of mouse intestinal organoid basal medium, mixed thoroughly, and brought to a final volume of 10 mL to prepare the mouse colonic organoid expansion medium. The medium was replaced every 2–3 days, and organoids were maintained at 37°C in a humidified incubator with 5% CO_2_. Organoid growth was monitored daily using an inverted microscope, and fully developed organoids typically appeared within 5–7 days. For experimental applications, organoids were passaged every 7–10 days. Organoids were dissociated into single cells using TrypLE Express (Gibco) and subsequently re‐embedded in fresh Matrigel to continue culture.

### 2.13 Surface Plasmon Resonance (SPR) Assay

SPR experiments were performed on a Biacore T200 system (GE Healthcare, Chicago, IL, USA) at 25°C. Recombinant human NMNAT1 protein was immobilized on a CM5 sensor chip using standard amine coupling, with a reference flow cell serving as control. Binding analyses were carried out in PBS‐P buffer at a flow rate of 30 µL/min. Serial dilutions of the Rhoifolin were injected to record association and dissociation phases, followed by regeneration with 10 mM glycine‐HCl (pH 2.5). Data were reference‐subtracted, solvent‐corrected, and globally fitted to a 1:1 binding model using Biacore Evaluation Software to determine kinetic parameters and equilibrium dissociation constants (KD).

### 2.14 Co‐immunoprecipitation Assay

As described previously, cells were transfected with plasmids according to standard protocols. After 48 h of transfection, cells were lysed in ice‐cold RIPA lysis buffer (P0013B, Beyotime, Shanghai, China) for 30 min with gentle rocking. Lysates were clarified by centrifugation at 12,000 rpm for 15 min at 4°C, and the protein concentration was determined using an enhanced BCA protein assay kit (P0010S, Beyotime, Shanghai, China). Equal amounts of protein were incubated with 2 µg of anti‐Flag (20543‐1‐AP, Proteintech, Wuhan, China), anti‐His (66005‐1‐lg, Proteintech, Wuhan, China) or control IgG (3900S, CST, Danvers, MA, USA) antibodies at 4°C overnight with constant rotation. Protein A/G agarose beads (BD0045, Biogot technology, Nanjing, China) were then added to the mixture and incubated for 2 h at 4°C. The beads were washed five times with RIPA lysis buffer, and bound proteins were eluted with loading buffer by heating at 100°C for 5 min. The samples were separated by SDS‐PAGE and analyzed by western blotting assay.

### 2.15 Small Interfering RNA (siRNA) and Plasmid Transfection

Cells were seeded in 6‐well plates at a density of 2 × 10^5^ cells per well and cultured overnight in complete growth medium to achieve a confluency of 60–80% on the day of transfection. The siRNA was purchased from Hanbio Biotechnology (Shanghai, China), and the siRNA sequences are listed in Supplementary Table S1. The wild‐type and mutant NMNAT1 constructs were obtained from Public Protein/Plasmid Library (PPL, Nanjing, China). For siRNA transfection, 50 nM siRNA was diluted in Opti‐MEM (31985062, Thermo Fisher Scientific, Waltham, MA, USA), while for plasmid DNA transfection, 2 µg of plasmid DNA was similarly diluted. Separately, 5 µL of Lipofectamine 3000 Reagent (L3000015, Thermo Fisher Scientific, Waltham, MA, USA) was diluted in Opti‐MEM. The siRNA or plasmid DNA was then mixed with the diluted lipofectamine, followed by a 15‐min incubation at room temperature to allow for the formation of transfection complexes. The prepared transfection complexes were added dropwise to cells in serum‐ and antibiotic‐free Opti‐MEM, and the cells were incubated at 37°C in a 5% CO_2_ humidified incubator for 4–6 h, after which the medium was replaced with fresh complete growth medium. Cells were cultured for an additional 24 h before performing downstream analyses to evaluate transfection efficiency and functional outcomes.

### 2.16 CUT&Tag

Chromatin was isolated from both treated and control cells using the CUT&Tag kit (TD904, Vazyme, Nanjing, China) according to the manufacturer's instructions. Briefly, cells were harvested and incubated with activated ConA beads for 10 min at room temperature, followed by magnetic separation. The bead‐bound cells were then incubated overnight at 4°C with the FOXO1 primary antibody (FOXO1, #2880, CST, Boston, MA, USA). On the following day, cells were washed and incubated with the corresponding secondary antibody for 45 min at room temperature. After additional washes, pA/G‐Tnp Pro transposase was added at a final concentration of 0.04 µM and incubated at room temperature for 1 h to facilitate the insertion of sequencing adapters into accessible chromatin regions. Chromatin fragmentation was subsequently achieved by incubation at 37°C for 60 min, enabling Tn5‐mediated cleavage at open chromatin sites. DNA was then extracted using DNA purification beads, washed to remove contaminants, and resuspended in ddH2O for storage at −20°C or immediate use in qPCR. The qPCR was performed using primers spanning sequential regions of the Il22 promoter to evaluate FOXO1 enrichment. Specifically, primers were designed to amplify ten overlapping fragments: *Il22*‐1 (−2000 to −1701 bp), *Il22*‐2 (‐1800 to −1501 bp), *Il22*‐3 (−1600 to −1301 bp), *Il22*‐4 (−1400 to −1101 bp), *Il22*‐5 (−1200 to −901 bp), *Il22*‐6 (−1000 to −701 bp), *Il22*‐7 (−800 to −501 bp), *Il22*‐8 (−600 to −301 bp), *Il22*‐9 (−400 to −101 bp), *Il22*‐10 (−200 to −1 bq) upstream of the transcription start site. All experiments were repeated four times independently, and the obtained data were normalized to that of the IgG control.

### 2.17 Measurement of NAD+ Levels

Measurement of NAD+ levels were quantified using a commercially available NAD/NADH assay kit (WST‐8 method, Beyotime, S0175, Shanghai, China) following the manufacturer's instructions. Briefly, cells in 6‐well plates were collected and lysed with NAD+/NADH extract on ice, and the supernatant was used as the sample to be tested. A control solution with a gradient concentration of NADH was prepared with a 10 mmol/L NADH standard. The supernatant from the sample was first aspirated and used to determine the total amount of NAD+ and NADH. Subsequently, the sample was heated at 60°C for 30 min to remove the NAD+, after which the supernatant was collected to determine the NADH concentration. Ethanol dehydrogenase was added to each well, and the plate was incubated at 37°C in the dark for 10 min. Then, 10 µL of chromogenic solution was added, and the plate was incubated for 30 min. Afterward, the absorbance at 450 nm was measured, and the content was calculated by comparison with the standard curve. The content of NAD+ was calculated by subtracting the content of NADH from the total amount.

### 2.18 Chemotaxis of ILC3s in Colitis Mice

ILC3s were isolated from the spleens of wild‐type donor mice and then cultured in RPMI 1640 medium supplemented with 10% FBS, 1% p/s, recombinant murine IL‐7 (20 ng/mL), IL‐2 (10 ng/mL), and IL‐15 (10 ng/mL) to maintain survival and proliferation. Cells were treated with or without Rhoifolin (30 µM) for 48 h. Prior to transfer, ILC3s were labeled with the fluorescent dye Dil (5 µM, 30 min, 37°C), washed extensively, and resuspended in sterile PBS. For adoptive transfer, recipient mice were pretreated with DSS to establish experimental colitis and then injected intravenously via the tail vein with 1 × 10^6^ Rhoifolin‐treated or untreated ILC3s per mouse. At day 5 after adoptive transfer, animals were sacrificed and colonic tissues were collected. Paraffin‐embedded sections were prepared, and immunofluorescence staining was performed to detect Dil‐labeled ILC3s in situ to confirm their identity. Fluorescent images were acquired using a confocal laser scanning microscope, and ILC3s localization within the colonic lamina propria was evaluated.

### 2.19 Statistical Analysis

Data are expressed as means ± standard error of the mean (S.E.M.). No data transformation or exclusion of outliers was performed unless otherwise stated. All statistical analyses were performed using GraphPad Prism version 9.0 (GraphPad Software, La Jolla, CA, USA). Comparisons among multiple groups were conducted using one‐way analysis of variance (ANOVA) followed by Tukey's post hoc test for multiple comparisons. All statistical tests were two‐sided where applicable. The *p* value < 0.05 was considered statistically significant. Statistical significance was defined as ^*^ *p* < 0.05, ^**^ *p* < 0.01. For animal studies, each experimental group generally consisted of six mice (n = 6). For in vitro experiments, data were obtained from at least three independent biological replicates unless otherwise specified. The sample size (n) for each experiment is indicated in the corresponding figure legends.

## 3 Results

### 3.1 Rhoifolin Restores Colonic Epithelial Barrier Integrity to Alleviate Colitis

To evaluate the therapeutic potential of Rhoifolin in experimental colitis, C57BL/6 mice were subjected to DSS‐induced colitis (Figure 1A). Rhoifolin treatment significantly attenuated body weight loss, reduced disease activity index (DAI) scores and preserved colon length compared with DSS‐treated mice (Figure 1B–D). Histopathological analysis further showed that Rhoifolin markedly alleviated DSS‐induced colonic injury, as evidenced by reduced inflammatory cell infiltration, improved crypt architecture, and attenuated epithelial damage (Figure 1E). In parallel, Rhoifolin (3, 10 mg/kg) markedly decreased colonic myeloperoxidase (MPO) activity and neutrophils infiltration in the colonic lamina propria (Figure 1F,G). Small‐animal endoscopic assessment further confirmed the protective effects of Rhoifolin, revealing markedly reduced mucosal inflammation (Figure 1H). This was accompanied by significantly decreased colonic expression of proinflammatory cytokines including *Tnf*, *Il1* and *Il6* (Figure 1I). The protective effects of Rhoifolin were further validated in both 2, 4, 6‐trinitrobenzenesulfonic acid (TNBS)‐induced and chronic DSS‐induced colitis models. In TNBS‐induced colitis, Rhoifolin significantly preserved colon length, reduced MPO activity, alleviated histopathological and macroscopic injury, and decreased neutrophil accumulation in the colonic lamina propria (Figure S1A–E). In the chronic colitis model, Rhoifolin similarly alleviated disease severity, as reflected by improved body weight recovery, preserved colon length, reduced MPO activity, inhibited neutrophil infiltration, ameliorated histological, and endoscopic injury (Figure S2A–F). It also suppressed mRNA expression of *Tnf*, *Il1*, and *Il6* in colons (Figure S2G). Together, these findings demonstrate that Rhoifolin confers broad protection across acute, chronic, and chemically distinct models of experimental colitis.

**FIGURE 1:**
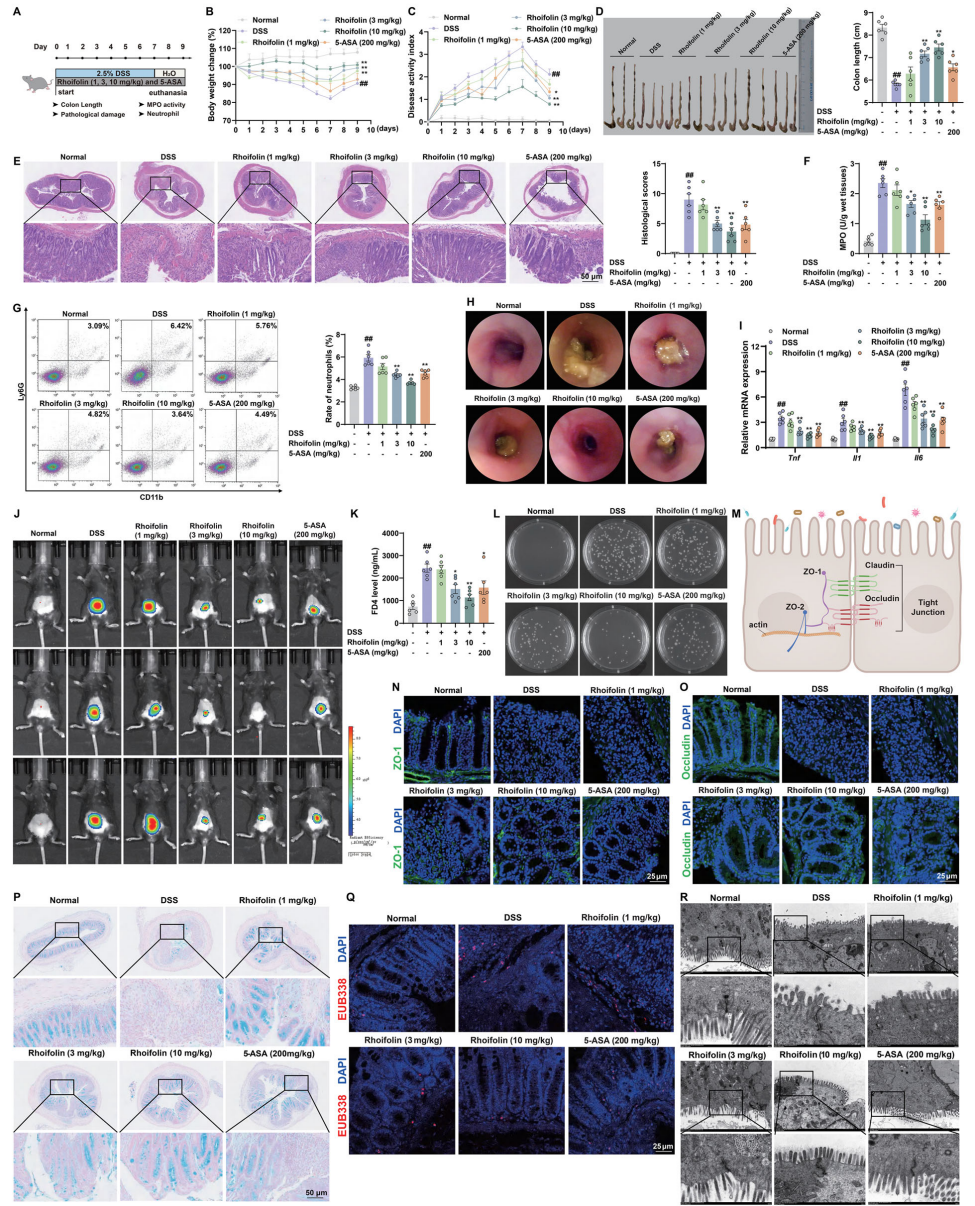
Rhoifolin restores colonic epithelial barrier integrity to alleviate colitis. (A) C57BL/6 mice were administrated 2.5% DSS to induce colitis and treated with Rhoifolin (1, 3, 10 mg/kg) throughout the experiment. (B) Body weight change. (C) DAI scores. (D) Representative colon images and colon length. (E) Representative H&E‐stained colon sections and histological scores. (F) MPO activity in colons. (G) Flow cytometric analysis of neutrophil infiltration in colonic lamina propria. (H) Representative endoscopic images of colons. (I) The mRNA levels of *Tnf*, *Il1*, and *Il6* in colons. (J, K) FD4 fluorescence distribution in the abdominal region and serum FD4 levels. (L) Bacterial load in spleens and mesenteric lymph nodes. (M) Schematic illustration of the intestinal barrier structure. (N, O) Representative immunofluorescence images of ZO‐1 and Occludin. (P) Representative Alcian blue‐stained colon sections. (Q) Representative FISH images using the EUB338 probe to visualize bacterial localization relative to the colonic epithelium. (R) Representative transmission electron microscopy images of colonic ultrastructure. Data were presented as means ± S.E.M of six mice in each group (n = 6). ^##^ *P* < 0.01 vs. Normal group. ^*^ *P* < 0.05, ^**^ *P* < 0.01 vs. DSS group.

The in vivo safety profile of Rhoifolin was subsequently assessed. Daily administration of Rhoifolin (1, 3, 10 mg/kg) for 30 consecutive days did not induce significant changes in body weight or colon length compared with vehicle‐treated controls (Figure S3A,B). Serum biochemical analyses revealed no detectable alterations in alanine aminotransferase (ALT), aspartate aminotransferase (AST), urea, or creatinine levels (Figure S3C,D). Histopathological examination of major organs, including heart, liver, spleen, lung, kidney, and colon, revealed no evidence of tissue injury or morphological abnormalities (Figure S3E). Furthermore, flow cytometric assessment of splenic immune cell populations including CD3^+^CD4^+^ T cells, CD3^+^CD8^+^ T cells, CD19^+^ B cells, CD11b^+^Ly6G^+^ neutrophils, CD11b^+^Ly6C^+^ monocytes and CD11b^+^F4/80^+^ macrophages showed no significant alterations in their frequencies following Rhoifolin treatment (Figure S3F–K). These findings indicate that Rhoifolin confers potent anti‐colitis activity without eliciting systemic toxicity or disrupting basal immune homeostasis.

To elucidate the mechanism underlying the protective effects of Rhoifolin, we next evaluated colonic epithelial barrier integrity. DSS‐treated mice exhibited significantly higher FD4 fluorescence in the abdominal cavity and serum, indicating enhanced intestinal permeability, whereas Rhoifolin (3, 10 mg/kg) substantially reduced FD4 leakage in a dose‐dependent manner (Figure 1J,K). Rhoifolin also limited DSS‐induced bacterial translocation to the spleen and mesenteric lymph nodes (MLNs) (Figure 1L). The intestinal epithelial barrier is a highly specialized defense structure composed of a continuous epithelial layer supported by organized villus architectures and intercellular tight junctions such as ZO‐1 and Occludin *etc*. (Figure 1M). Immunofluorescence staining revealed that Rhoifolin markedly restored the disrupted localization and continuity of ZO‐1 and Occludin induced by DSS challenge (Figure 1N,O). Alcian blue staining further demonstrated a substantial depletion of goblet cells and reduced mucin production following DSS exposure, which was markedly improved by Rhoifolin (Figure 1P). In addition, FISH analysis using the universal bacterial probe EUB338 showed that Rhoifolin effectively prevented bacterial encroachment toward the epithelial surface (Figure 1Q). Transmission electron microscopy (TEM) analysis further confirmed that Rhoifolin preserved villus morphology, maintained intact tight junction structures, and prevented DSS‐induced widening of intercellular spaces (Figure 1R). Collectively, these findings indicate that Rhoifolin protects against colitis primarily by maintaining intestinal epithelial barrier integrity.

### 3.2 Rhoifolin Promotes IL‐22 Production to Restore Epithelial Barrier Integrity

To determine whether Rhoifolin exerts direct protective effects on intestinal epithelial cells under inflammatory conditions, we first established a TNF‐α induced epithelial injury model using polarized Caco‐2 monolayers and evaluated barrier integrity by TEM and immunofluorescence assays. Direct exposure to Rhoifolin (1, 3, 10, 30 µM) did not induce discernible improvements in epithelial ultrastructure or restore expression and localization of tight junction proteins ZO‐1 and Occludin (Figure S4A–C). To further evaluate epithelial‐intrinsic effects in a physiologically relevant system, we established a murine colonic organoid culture system that recapitulates the three‐dimensional architecture of the intestinal epithelium (Figure S4D). Consistently, Rhoifolin failed to rescue DSS‐induced structural collapse or tight junction disruption in colonic organoids (Figure S4E–G). These results suggest that Rhoifolin does not directly reinforce epithelial barrier integrity in isolated epithelial models. Instead, its barrier‐protective effects observed in vivo are likely mediated indirectly through the modulation of soluble mediators such as cytokines.

IL‐22 is a key cytokine that maintains intestinal epithelial barrier integrity by promoting epithelial regeneration, enhancing tight junction formation, and stimulating antimicrobial peptide expression. Notably, Rhoifolin treatment led to a significant upregulation of colonic IL‐22 expression compared with the DSS group (Figure 2A,B). To directly ascertain whether IL‐22 is essential for the anti‐colitis activity of Rhoifolin, an IL‐22‐neutralizing antibody was co‐administered in mice. Strikingly, IL‐22 neutralization almost completely abolished the therapeutic benefits of Rhoifolin, as evidenced by the reversal of improvements in DAI scores, colon length, endoscopic appearance, neutrophil infiltration, colonic MPO activity, and histopathological damage (Figure 2C–H). Consistently, blockade of IL‐22 eliminated the epithelial barrier‐protective effects conferred by Rhoifolin. Specifically, bacterial translocation to the spleen and MLNs was restored, and in vivo permeability imaging revealed a marked increase in intestinal leakiness following IL‐22 neutralization (Figure 2I,J). The TEM further demonstrated re‐disruption of tight junction ultrastructure, while FISH analysis demonstrated renewed bacterial encroachment toward the epithelium (Figure 2K,L). Moreover, the Rhoifolin‐induced enhancement of ZO‐1, Occludin expression and continuity was markedly diminished upon IL‐22 blockade (Figure 2M,N). Together, these findings establish IL‐22 as an indispensable mediator of the anti‐colitis and epithelial barrier‐preserving actions of Rhoifolin.

**FIGURE 2:**
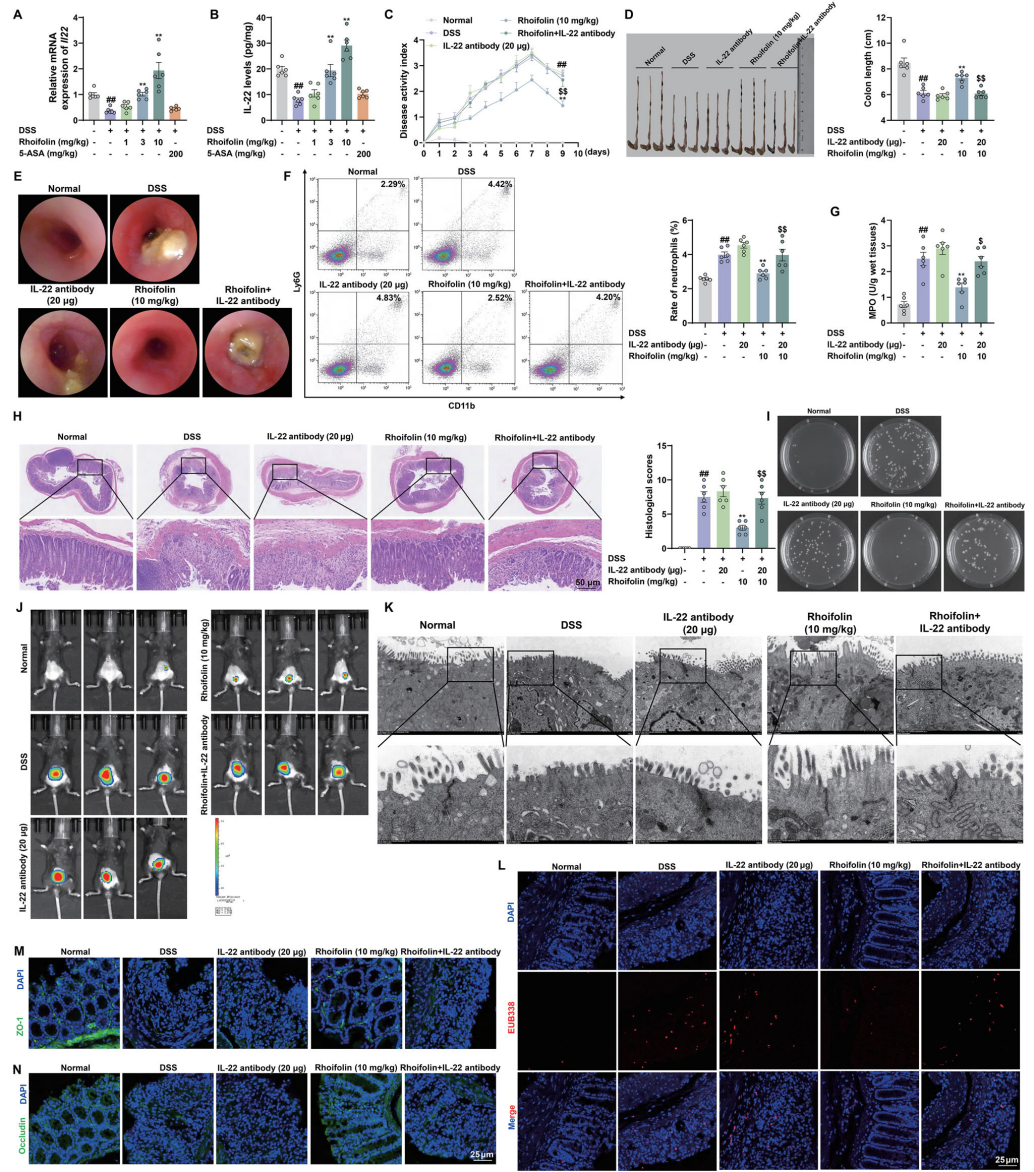
Rhoifolin promotes IL‐22 production to restore epithelial barrier integrity. (A, B) Female C57BL/6 mice were administered 2.5% DSS to induce colitis and concurrently treated with Rhoifolin (1, 3, 10 mg/kg) for 10 consecutive days. IL‐22 mRNA and protein levels in colons were assessed by qPCR and ELISA, respectively. (C‐N) Female C57BL/6 mice were subjected to DSS‐induced colitis model. Rhoifolin (10 mg/kg) was given once daily by oral gavage throughout the 10‐day experimental period. IL‐22 neutralizing antibody (20 µg/mouse) was intraperitoneal injected on days ‐1, 0, 3, 6, 9 relative to DSS treatment. DAI scores (C); representative colon images and colon length (D); representative endoscopic images (E); infiltration of neutrophils in colonic lamina propria (F); MPO activity in colons (G); representative H&E‐stained colon sections and histological scores (H); bacterial load in spleens and mesenteric lymph nodes (I); distribution and fluorescence intensity of FD4 in abdominal region (J); representative transmission electron microscopy images of colons (K); representative FISH images using the EUB338 probe to visualize bacterial localization relative to the colonic epithelium (L); representative immunofluorescence images of ZO‐1 and Occludin (M, N). Data were presented as means ± S.E.M of six mice in each group (n = 6). ^##^ *P* < 0.01 vs. Normal group. ^**^ *P* < 0.01 vs. DSS group. ^$^ *p* < 0.05, ^$$^ *p* < 0.01 vs. Rhoifolin group.

### 3.3 Rhoifolin Enhances ILC3s Effector Function to Promote IL‐22 Generation

IL‐22 is predominantly produced by ILC3s and CD4^+^ T cells in the intestinal mucosa [16, 17]. To determine whether CD4^+^ T cells are required for the anti‐colitis effects of Rhoifolin, DSS‐challenged mice were treated with a CD4^+^ T cell‐neutralizing antibody. Remarkably, depletion of CD4^+^ T cells did not compromise the therapeutic benefits of Rhoifolin, which still effectively alleviated body weight loss, reduced DAI scores, preserved colon length, decreased colonic MPO activity, improved histopathological damage and suppressed neutrophil infiltration in colonic lamina propria (Figure 3A–F). Correspondingly, epithelial barrier protection, as assessed by in vivo intestinal permeability imaging, remained largely intact following CD4^+^ T cell depletion (Figure 3G). We next investigated whether ILC3s directly contribute to Rhoifolin‐mediated protection. The mice received adoptive transfer of either Rhoifolin‐treated or untreated ILC3s (R‐ILC3s vs. ILC3s). Strikingly, mice receiving Rhoifolin‐conditioned ILC3s (R‐ILC3) exhibited substantially enhanced protection against DSS‐induced colitis compared with those receiving untreated ILC3s, reflected by enhanced body weight maintenance, reduced DAI scores, preserved colon length, decreased MPO activity, lower histopathological scores, and diminished neutrophil infiltration (Figure 3H–M). Intestinal barrier integrity was similarly improved in mice accepted Rhoifolin‐ILC3s (R‐ILC3s), demonstrated by reduced intestinal permeability in small animal imaging and lower serum FD4 concentrations (Figure 3N,O). These findings indicate that the therapeutic and barrier‐protective effects of Rhoifolin are predominantly mediated by ILC3s regulation.

**FIGURE 3:**
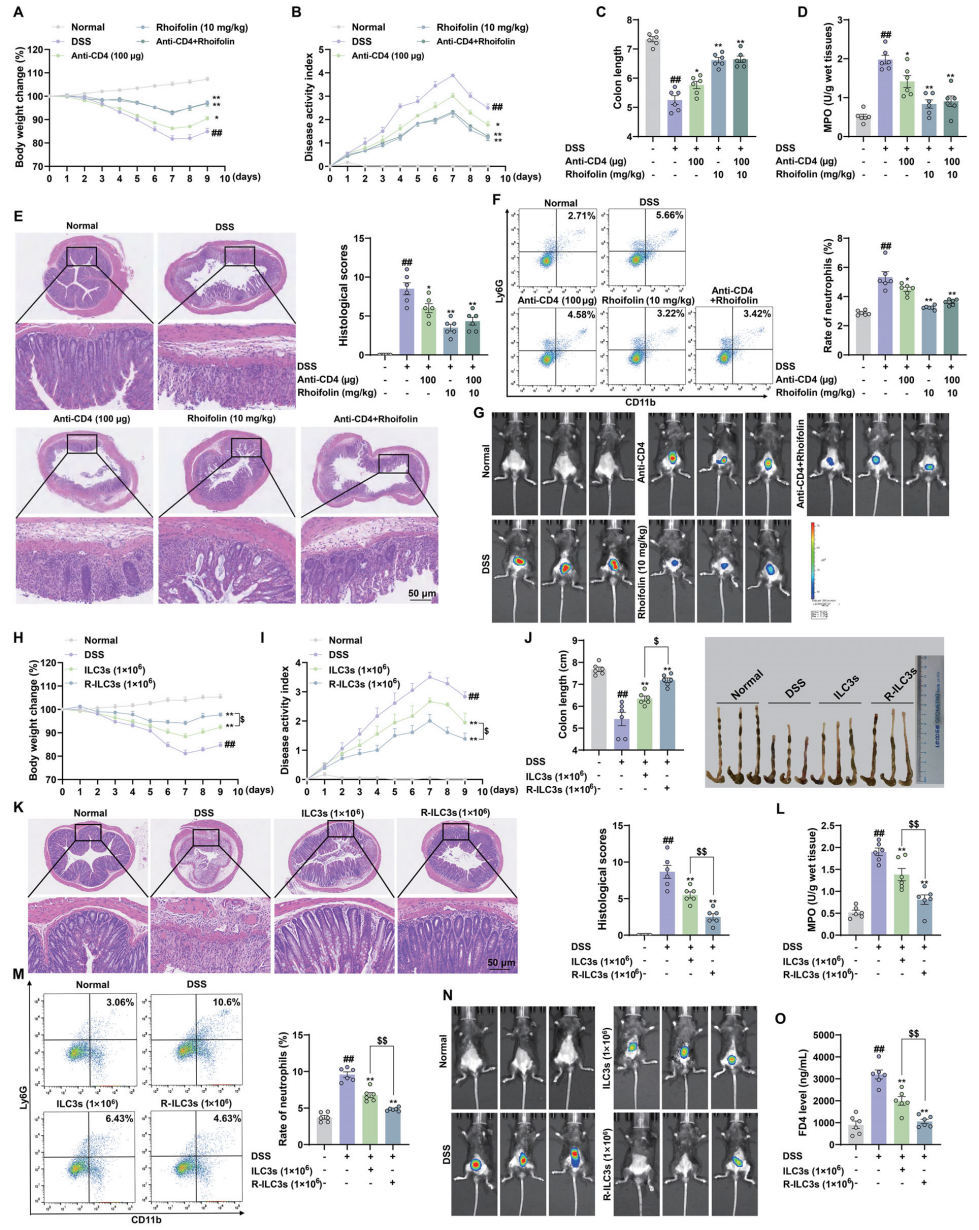
Rhoifolin alleviates colitis by targeting ILC3s. (A–G) Female C57BL/6 mice were subjected to a DSS‐induced colitis model. Rhoifolin (10 mg/kg) was given once daily by oral gavage throughout the 10‐day experimental period. CD4 monoclonal antibody (100 µg/mouse) was intraperitoneal injected on days ‐3, 0, 3 relative to DSS treatment. The body weight change (A); DAI scores (B); colon length (C); MPO activity in colons (D); representative H&E‐stained colon sections and histological scores (E); flow cytometric analysis of neutrophil infiltration in colonic lamina propria (F); distribution and fluorescence intensity of FD4 in abdominal region (G). (H‐O) The female C57BL/6 received either Rhoifolin‐treated (defined as R‐ILC3s) or untreated ILC3s (defined as ILC3s) on days ‐2, 2, 5 relative to DSS treatment. Body weight change (H); DAI scores (I); representative colon images and colon length (J); representative H&E‐stained colon sections and histological scores (K); MPO activity (L); flow cytometric analysis of neutrophil infiltration in colonic lamina propria (M); In vivo imaging of intestinal permeability (N); serum FD4 levels (O). Data were presented as mean ± S.E.M of six mice per group (n = 6). ^##^ *p* < 0.01 vs. Normal group; ^*^ *p* < 0.05, ^**^ *p* < 0.01 vs. DSS group; ^$^ *p* < 0.05, ^$$^ *p* < 0.01 vs. ILC3s group.

To elucidate how Rhoifolin elevated IL‐22 levels in colonic ILC3s, several mechanisms were considered, including alterations in ILCs lineage composition, expansion or reduced apoptosis of ILC3s, enhanced recruitment of ILC3s to the colon, improvement of ILC3s development, or direct augmentation of ILC3s effector function. Rhoifolin did not markedly alter the proportions of colonic ILC1s or ILC2s (Figure S5A), nor did it change the distribution of major ILC3s subsets, including CCR6^+^NKp46^−^ LTi‐like ILC3s, CCR6^−^NKp46^+^ NCR^+^ ILC3s, and CCR6^−^NKp46^−^ double‐negative ILC3s (Figure S5B). In addition, Rhoifolin had no obvious effect on ILC3s viability, proliferation, or apoptosis (Figure S5C–E). Chemotaxis assays revealed comparable infiltration of intravenously administered Rhoifolin‐treated and untreated ILC3s into the colonic lamina propria, and expression levels of chemokine receptors including CCR9 and CCR6 remained unchanged (Figure S5F–H). Furthermore, Rhoifolin did not alter bone marrow ILC‐related progenitor populations, including αLPs, defined as live Lin^−^CD127^+^c‐kit^+^ cells, and ILCPs (Figure S5I). These data indicate that Rhoifolin‐induced IL‐22 upregulation is unlikely to result from broad remodeling of the ILC compartment, selective expansion of specific ILC3s subsets, altered ILC3s survival or recruitment, or enhanced ILCs developmental output.

We therefore asked whether Rhoifolin directly enhanced the effector function of existing ILC3s. ILC3s isolated from the colonic lamina propria were treated with Rhoifolin *ex vivo*. Rhoifolin significantly increased *Il22* mRNA expression in these cells, whereas *Il17a* mRNA expression was not markedly altered (Figure 4A,B). Consistently, ELISA demonstrated a marked increase in IL‐22 secretion in the culture supernatants following Rhoifolin treatment (Figure 4C), confirming that Rhoifolin directly enhances IL‐22‐producing effector function of ILC3s at both the transcriptional and protein levels. To determine the functional consequence of this activation, conditioned medium from Rhoifolin‐treated ILC3s was applied to a TNF‐α induced Caco‐2 epithelial injury model. Exposure to this medium markedly improved epithelial barrier integrity, as indicated by increased transepithelial electrical resistance (TEER), restoration of intercellular junctions and microvillus architecture under TEM, and enhanced immunofluorescence staining of ZO‐1 and Occludin (Figure 4D–G). Similarly, in a three‐dimensional colonic organoid system, co‐culture with Rhoifolin‐conditioned ILC3s for 48 h significantly restored organoid morphology following DSS‐induced injury and enhanced expression, continuity of ZO‐1 and Occludin (Figure 4H–K). Taken together, these findings collectively demonstrate that Rhoifolin preserves intestinal epithelial barrier integrity by directly enhancing ILC3s effector function and IL‐22 production. Notably, the molecular mechanism underlying ILC3s effector function enhancement remain largely unexplored, and pharmacological strategies, particularly small molecule approaches, capable of precisely modulating ILC3s effector function are exceedingly scare. Therefore, we next employed Rhoifolin as a functional chemical probe to dissect the previously unrecognized intrinsic mechanisms governing ILC3s effector functional enhancement.

**FIGURE 4:**
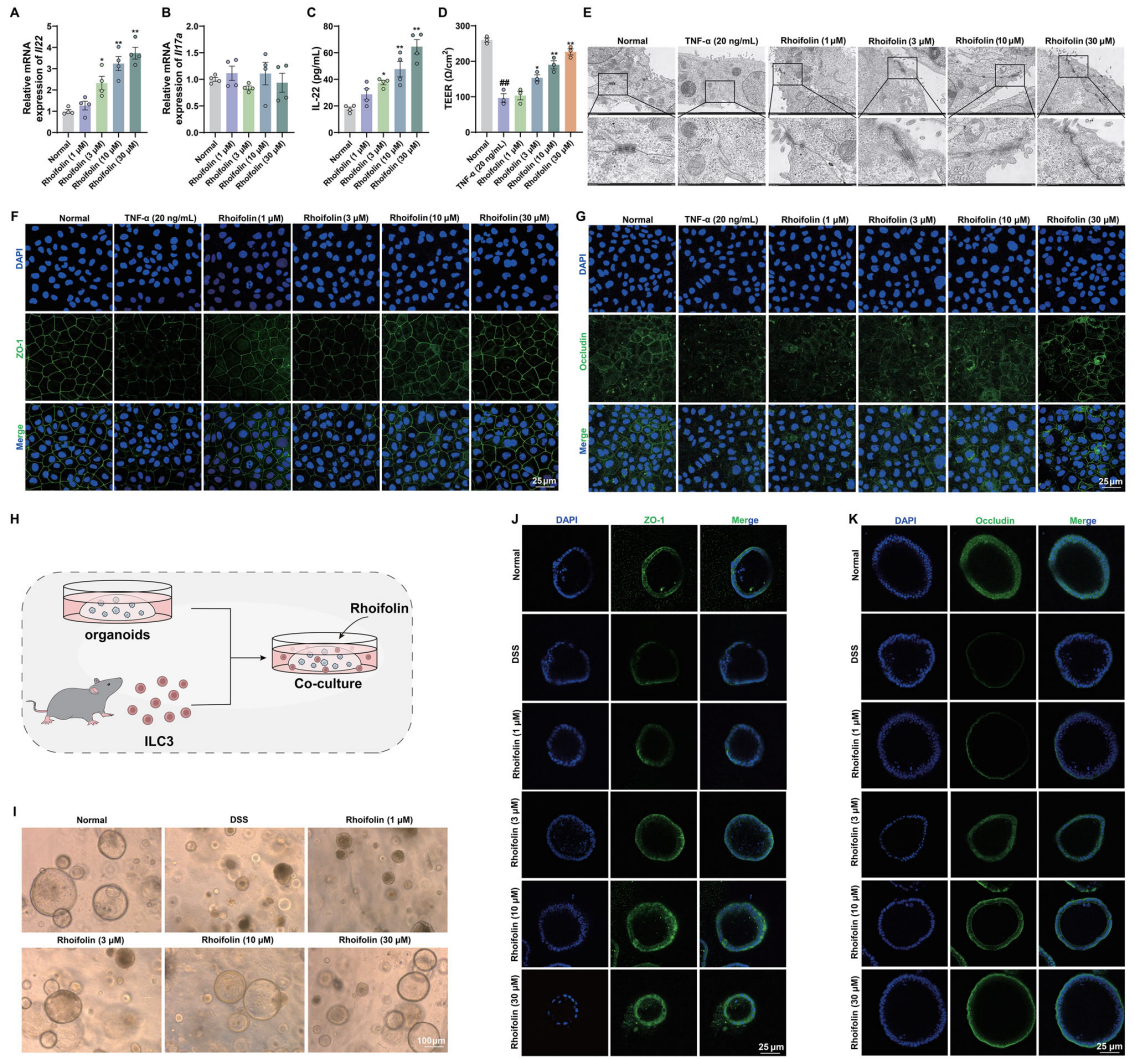
Rhoifolin enhances ILC3s effector function to guarantee IL‐22 production. (A–C) ILC3s isolated from the colonic lamina propria of mice were treated with Rhoifolin (1, 3, 10, 30 µM) for 48 h. The *Il22* and *Il17a* mRNA expression was measured by qPCR assay (A, B); IL‐22 secretion in supernatants was measured by ELISA (C). (D–G) Caco‐2 cells were subjected to TNF‐α induced injury, followed by treatment with conditioned medium from either Rhoifolin‐cultured ILC3s, the TEER was detected on day 21 (D); representative transmission electron microscopy images of Caco‐2 cells (E); representative immunofluorescence images of ZO‐1 and Occludin in Caco‐2 cells (F, G). (H–K) Colonic organoids were co‐cultured with ILC3s, followed by DSS stimulation and Rhoifolin treatment (H); morphological change of colonic organoids (I); representative immunofluorescence images of ZO‐1 and Occludin in colonic organoids (J, K). Data were presented as mean ± S.E.M from three independent in vitro experiments (n = 3). ^*^ *p* < 0.05, ^**^ *p* < 0.01 vs. Normal group.

### 3.4 Rhoifolin Enhances ILC3s Effector Function Through the Nicotinamide Salvage Pathway

To elucidate the mechanisms underlying Rhoifolin‐mediated enhancement of ILC3s effector function, we performed RNA‐seq analysis of purified ILC3s treated with or without Rhoifolin. Transcriptomic profiling identified 235 upregulated and 72 downregulated genes after Rhoifolin treatment using the thresholds of (|log_2_FC| > 1, *p* < 0.05) (Figure 5A). Reactome pathway enrichment analysis revealed several altered biological pathways, among which the nicotinamide salvage pathway was significantly enriched and ranked seventh (Figure 5B). Consistently, untargeted metabolomic profiling identified nine significantly altered metabolites in Rhoifolin‐treated ILC3s (Figure 5C, Table S2), among which 1‐methylnicotinamide was annotated to nicotinamide metabolism (Figure 5D). The convergence of these transcriptomic and metabolomic findings pointed to nicotinamide salvage metabolic axis as a candidate mechanism underlying Rhoifolin‐enhanced ILC3s effector function.

**FIGURE 5:**
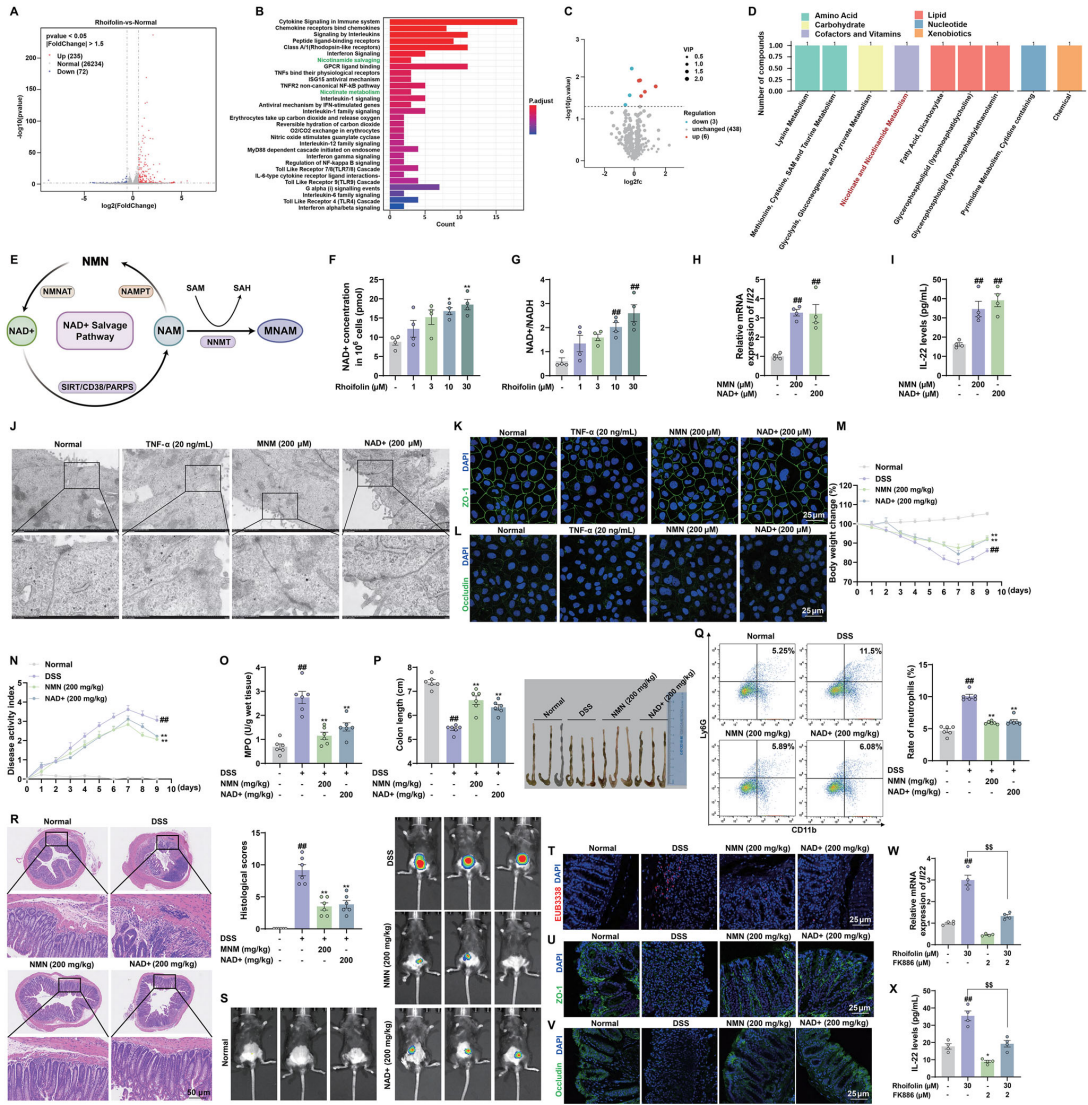
Rhoifolin promotes nicotinamide salvage pathway to enhance ILC3s effector function. (A, B) Volcano plot and Reactome enrichment analysis of differentially expressed genes in Rhoifolin‐treated ILC3s. (C, D) Volcano plot and pathway enrichment analysis of altered metabolites in Rhoifolin‐treated ILC3s. (E) Schematic illustration of the nicotinamide salvage pathway. (F, G) Intracellular NAD+ levels and NAD+/NADH ratio in ILC3s following Rhoifolin treatment. (H, I) ILC3s isolated from the intestinal lamina propria of mice were treated with nicotinamide (NAM) or NAD+ for 48 h, the mRNA and protein levels of IL‐22 were detected by qPCR and ELISA, respectively. (J–L) Representative transmission electron microscopy and immunofluorescence images of ZO‐1 and Occludin in Caco‐2 cells cultured with conditioned media from NAM‐ or NAD+‐treated ILC3s. (M‐V) Female C57BL/6 mice were subjected to DSS‐induced colitis. NAM (100 mg/kg) or NAD+ (100 mg/kg) were given once daily by oral gavage throughout the 10‐day experimental period, and the body weight change was calculated (M); DAI scores (N); MPO activity (O); colon length (P); flow cytometry analysis of neutrophil infiltration in colonic lamina propria (Q); representative H&E staining of colons (R); distribution and fluorescence intensity of FD4 in abdominal region (S); representative FISH images using the EUB338 probe to visualize bacterial localization relative to the colonic epithelium (T); representative immunofluorescence images of ZO‐1 and Occludin in colon tissues (U, V). (W, X) ILC3s isolated from intestinal lamina propria of mice were pretreated with FK866 for 2 h, and then cultured with Rhoifolin for 48 h, mRNA and protein levels of IL‐22 was detected by qPCR and ELISA, respectively. Data were presented as mean ± S.E.M from three independent in vitro experiments and six mice per group in vivo experiments (n = 6 in vivo and n = 3 in vitro experiments). ^#^ *p* < 0.05, ^##^ *p* < 0.01* vs*. Normal group; ^*^ *p* < 0.05, ^**^ *p* < 0.01* vs*. DSS group. ^$$^ *p* < 0.01 vs. Rhoifolin group.

Given that NAD+ is terminal product of the nicotinamide salvage pathway, where nicotinamide is sequentially converted by phosphoribosyltransferase (NAMPT) and nicotinamide mononucleotide adenylyltransferase (NMNAT) enzymes [18], we directly quantified intracellular NAD+ levels (Figure 5E). Rhoifolin (1, 3, 10, 30 µM) induced a dose‐dependent upregulation of NAD+ content and markedly increased the NAD+/NADH ratio (Figure 5F,G), reinforcing the hypothesis that Rhoifolin enhances ILC3s effector function through activation of nicotinamide salvage pathway. Since the involvement of nicotinamide salvage pathway in regulating ILC3s effector function has not been previously reported, we next investigated the causal relationship between this pathway and IL‐22 production. Treatment of isolated ILC3s with either nicotinamide (NAM, the physiological precursor) or NAD+ (the terminal product) significantly increased IL‐22 mRNA and protein expression (Figure 5H,I). Conditioned medium from NAM‐ or NAD+‐treated ILC3s effectively preserved intestinal epithelial barrier integrity, as evidenced by preserved microvilli morphology, intact intercellular junctions under TEM, along with restored ZO‐1 and Occludin expressions by immunofluorescence staining (Figure 5J–L). We further evaluated the in vivo relevance of these findings in DSS‐induced colitis mice. Administration of NAM or NAD+ markedly alleviated body weight loss, reduced DAI scores, decreased MPO activity, preserved colon length, limited neutrophil infiltration in the colonic lamina propria, and mitigated histopathological damage (Figure 5M–R). Consistently, both treatments remarkably restored epithelial barrier function, as evidenced by reduced FD4 leakage, decreased bacterial translocation and enhanced protein expression of ZO‐1, Occludin in colons (Figure 5S–V). Collectively, these findings indicate that nicotinamide salvage pathway promotes IL‐22 production, supports ILC3s effector function, and contributes to epithelial barrier restoration, thereby ameliorating colitis.

Finally, to determine whether the nicotinamide salvage pathway is indispensable for the protective effects of Rhoifolin, we employed FK866, a potent inhibitor of nicotinamide salvage. Pharmacological inhibition of this pathway largely abrogated Rhoifolin‐induced enhancement of IL‐22 production (Figure 5W,X), demonstrating that Rhoifolin exerts its protective effects by activating the nicotinamide salvage pathway, thereby supporting ILC3s effector function and epithelial barrier integrity, ultimately mitigating colitis.

### 3.5 Rhoifolin Directly Targets NMNAT1 to Enhance Nicotinamide Salvage Pathway and ILC3s Effect Function

Having established that Rhoifolin promotes nicotinamide salvage metabolism to enhance ILC3s effector function, we next investigated the molecular mechanism underlying this metabolic reprogramming. Since the nicotinamide salvage pathway is primarily regulated by NAMPT and NMNAT enzymes [19], we first examined the expression profile of *Nmnat* isoforms in primary ILC3s. Among the three isoforms, *Nmnat1* was dominantly expressed, whereas *Nmnat2* and *Nmnat3* were presented at substantially lower levels (Figure S6A). Based on this expression pattern, we selected NMNAT1 as the major NMNAT isoform for subsequent mechanistic investigation, while also examining NAMPT as another key enzyme in the nicotinamide salvage pathway. The cellular thermal shift assay (CETSA) and solvent‐induced precipitation (SIP) assays revealed that Rhoifolin significantly increased the stability of NMNAT1, whereas NAMPT remained unaffected, indicating a direct interaction with NMNAT1 (Figure 6A,B). Further concentration‐gradient CETSA and SIP analyses confirmed dose‐dependent stabilization of NMNAT1 by Rhoifolin (Figure 6C,D). Surface plasmon resonance (SPR) analysis demonstrated a strong binding affinity (KD = 2.967 µM, Figure 6E), and in vitro enzymatic assays showed that Rhoifolin markedly enhanced NMNAT1 catalytic activity (Figure 6F).

**FIGURE 6:**
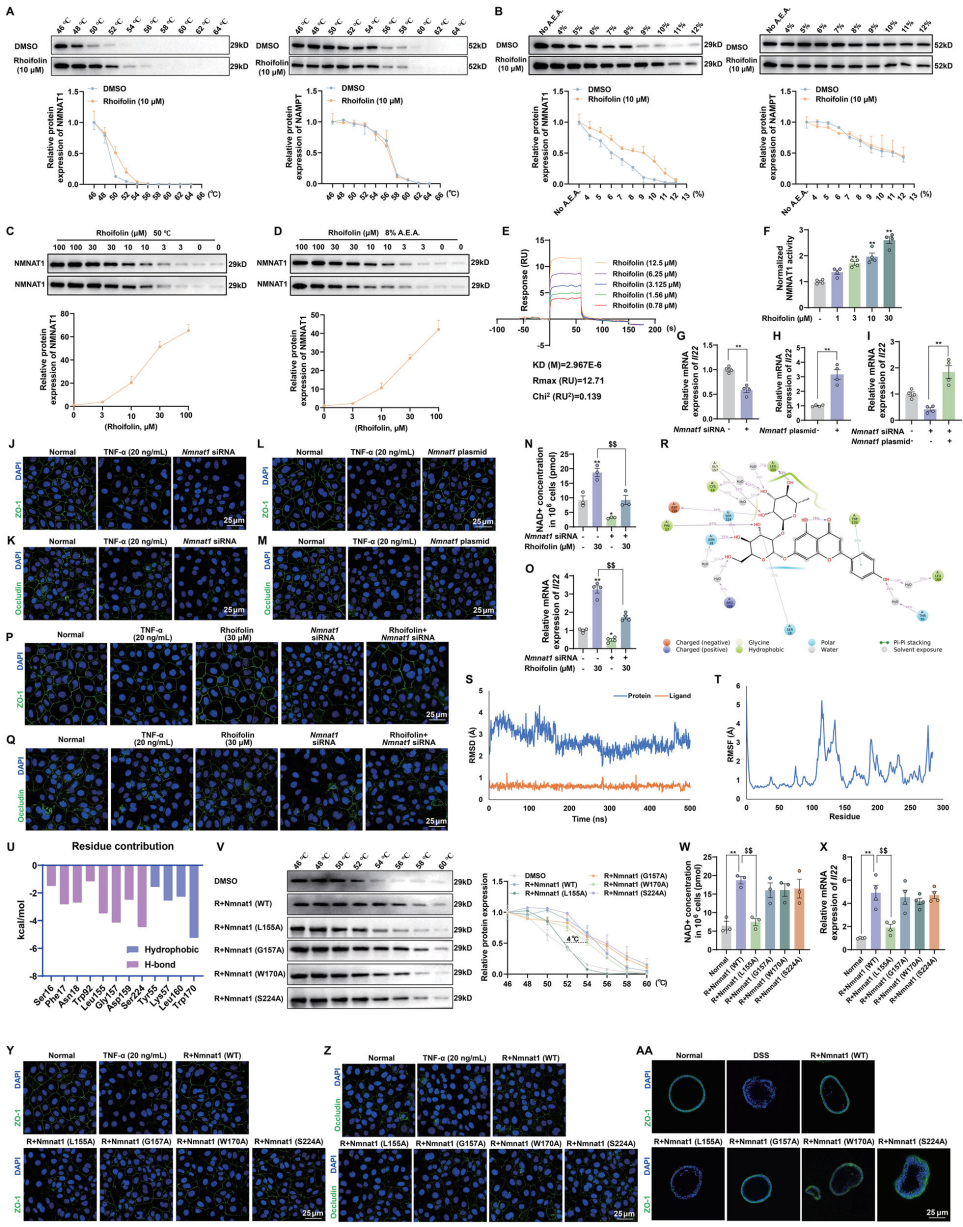
Rhoifolin targets NMNAT1 to facilitate nicotinamide salvage pathway. (A, B) CETSA and SIP assays assessing stability of NMNAT1 and NAMPT proteins in cell lysates treated with Rhoifolin. (C, D) Dose‐dependent CETSA and SIP assays evaluating thermal stability of NMNAT1 protein upon increasing concentrations of Rhoifolin. (E) SPR analysis measuring binding affinity between Rhoifolin and NMNAT1 protein. (F) Enzymatic activity of NMNAT1 was measured in vitro using Rhoifolin. (G–M) The ILC3s were transfected with *Nmnat1* siRNA or *Nmnat1* plasmid or *Nmnat1* siRNA+*Nmnat1* plasmid, and mRNA expression of *Il22* was detected by qPCR assay (G–I); the conditioned medium from *Nmnat1* siRNA or *Nmnat1* plasmid‐transfected ILC3s was applied to caco‐2 monolayers subjected to TNF‐α stimulation, and the protein expression of ZO‐1 and Occludin was analyzed by immunofluorescence assay (J‐M). (N–Q) ILC3s were transfected with *Nmnat1* siRNA, followed by Rhoifolin treatment for 48 h. The NAD+ level was detected by kits (N); mRNA expression of *Il22* was determined by qPCR assay (O); the conditioned medium from these ILC3s was applied to caco‐2 monolayers subjected to TNF‐α stimulation, and protein expression of ZO‐1 and Occludin was analyzed by immunofluorescence assay (P, Q). (R) Molecular docking showing key interactions between Rhoifolin and NMNAT1. (S, T) Molecular dynamics simulation analyses showing stable NMNAT1‐Rhoifolin complex. (U) Binding free energy decomposition identifying Trp170, Ser224, Gly157, and Leu155 as critical residues contributing to Rhoifolin‐NMNAT1 interaction. (V) CETSA was performed in HEK‐293T cells expressing wild‐type or Trp170, Ser224, Gly157, Leu155‐mutant NMNAT1 to evaluate Rhoifolin‐induced thermal stabilization. (W–Z) Functional rescue experiment was performed in NMNAT1‐deficient ILC3s reconstituted with either wild‐type or Leu155‐mutant NMNAT1, followed by Rhoifolin treatment for 48 h. The NAD+ level and NAD+/NADH ratio was determined by kits (W); mRNA expression of *Il22* was detected by qPCR assay (X); conditioned medium from these ILC3s were cultured with caco‐2 cells subjected to TNF‐α stimulation, and protein expression of ZO‐1 and Occludin was detected by immunofluorescence assay (Y, Z). (AA) The NMNAT1‐deficient ILC3s were reconstituted with either wild‐type or Leu155‐mutant NMNAT1, followed by cultured with colonic organoids. After culture with Rhoifolin (30 µM) for 48 h, protein expression of ZO‐1 was detected by immunofluorescence assay. Data were presented as mean ± S.E.M. from three independent in vitro experiments (n = 3). ^*^ *P* < 0.05, ^**^ *P* < 0.01 vs. Normal group; ^$^ *p* < 0.05, ^$$^ *p* < 0.01 vs. Rhoifolin or R+Nmnat1 (WT) group.

Because NMNAT1 has not previously been implicated in ILC3s biology, we next examined its functional relevance. Knockdown of NMNAT1 markedly decreased *Il22* transcription, whereas overexpression significantly increased *Il22* mRNA expression (Figure 6G,H). Importantly, re‐expression of NMNAT1 in *Nmnat1*‐deficient ILC3s restored *Il22* mRNA to levels comparable to or slightly exceeding those observed in untreated control cells, confirming the specificity of NMNAT1 in regulating *Il22* transcription (Figure 6I). Conditioned medium collected from *Nmnat1*‐silenced ILC3s effectively disrupted epithelial barrier integrity in Caco‐2 cells, as evidenced by decreased and intermittent expression of ZO‐1 and Occludin, whereas medium from NMNAT1‐overexpressing cells enhanced tight junction protein expression (Figure 6J–M). Consistently, *Nmnat1* knockdown substantially diminished Rhoifolin‐enhanced NAD+ generation, IL‐22 expression and its ability to promote intestinal barrier repair (Figure 6N–Q). By comparison, knockdown of *Nmnat2* or *Nmnat3* had minimal effects on Rhoifolin‐induced *Il22* mRNA expression in ILC3s (Figure S6B–D). Together, these findings highlighting the necessity of NMNAT1 for Rhoifolin‐enhanced nicotinamide salvage metabolism and ILC3s effector function.

To gain mechanistic insight into how Rhoifolin engages NMNAT1, we performed computational modeling and molecular dynamics (MD) simulations. The molecular docking predicted that Rhoifolin formed *π*–*π* stacking with Trp170 and hydrogen bonds with Leu155, Gly157, and Ser224 (Figure 6R). A 500 ns MD simulation confirmed that the NMNAT1‐Rhoifolin complex remained stable throughout the trajectory, with key residues exhibiting minimal atomic fluctuation less than 1.0 Å (Figure 6S,T). Binding free energy calculations using the MM/GBSA method revealed that van der Waals forces (∆Evdwa, −37.80 kcal/mol), electrostatic interactions (∆Eeleb, −52.70 kcal/mol), and non‐polar solvation energy (∆EGAd, −22.62 kcal/mol) collectively contributed to an overall binding free energy (∆Ebinde) of −99.36 kcal/mol (Supplementary Table S3). Specifically, four residues (Trp170, −5.23 kcal/mol, Ser224, −4.47 kcal/mol; Gly157, −4.13 kcal/mol; Leu155, −3.47 kcal/mol) were recognized as critical contributors, with hydrogen bonding and *π*–*π* stacking interactions playing dominant roles in stabilizing the complex (Figure 6U).

To validate the functional importance of these residues, wild‐type and site‐specific NMNAT1 mutants were transfected into HEK‐293T cells. CETSA analysis showed that mutation of Leu155 substantially weakened the interaction between Rhoifolin and NMNAT1 (Figure 6V). In ILC3s, reconstitution with the Leu155‐mutant NMNAT1 after endogenous knockdown markedly impaired Rhoifolin‐induced IL‐22 production and largely abolished its barrier‐restoring activity (Figure 6W‐AA). Collectively, these findings identify Leu155 as a pivotal residue mediating the direct interaction of Rhoifolin with NMNAT1, which is essential for promoting ILC3s‐derived IL‐22 production and subsequent intestinal barrier protection.

### 3.6 Rhoifolin Enhances ILC3s Effector Function via the SIRT1‐FOXO1 Axis

Building on our findings that NMNAT1‐mediated nicotinamide salvage metabolism is essential for Rhoifolin‐enhanced ILC3s effector function, we next explored the downstream mechanism linking this pathway to IL‐22 expression. Because NAD+ is an essential cofactor for sirtuin deacetylases [20], we first assessed SIRT family members potentially involved in this process. Database analysis revealed that SIRT1, SIRT3, SIRT5 and SIRT6 were implicated in UC pathogenesis (Figure 7A). Rhoifolin did not markedly alter the mRNA expression of *Sirt1‐7* in ILC3s (Figure 7B). Among these members, *Sirt1* exhibited the highest basal expression in ILC3s (Figure 7C), and Rhoifolin selectively enhanced SIRT1 enzymatic activity (Figure 7D). To determine whether SIRT1 is functionally required for Rhoifolin‐mediated ILC3s effector function enhancement, we used pharmacological inhibition and siRNA‐mediated knockdown approaches. The selective SIRT1 inhibitor EX527 markedly attenuated Rhoifolin‐induced *Il22* mRNA expression and IL‐22 production in ILC3s (Figure 7E,F). Consistently, *Sirt1* knockdown substantially impaired the ability of Rhoifolin to increase *Il22* transcription and IL‐22 protein secretion (Figure 7G,H). These results indicate that SIRT1 is required for Rhoifolin‐mediated enhancement of IL‐22 production in ILC3s.

**FIGURE 7:**
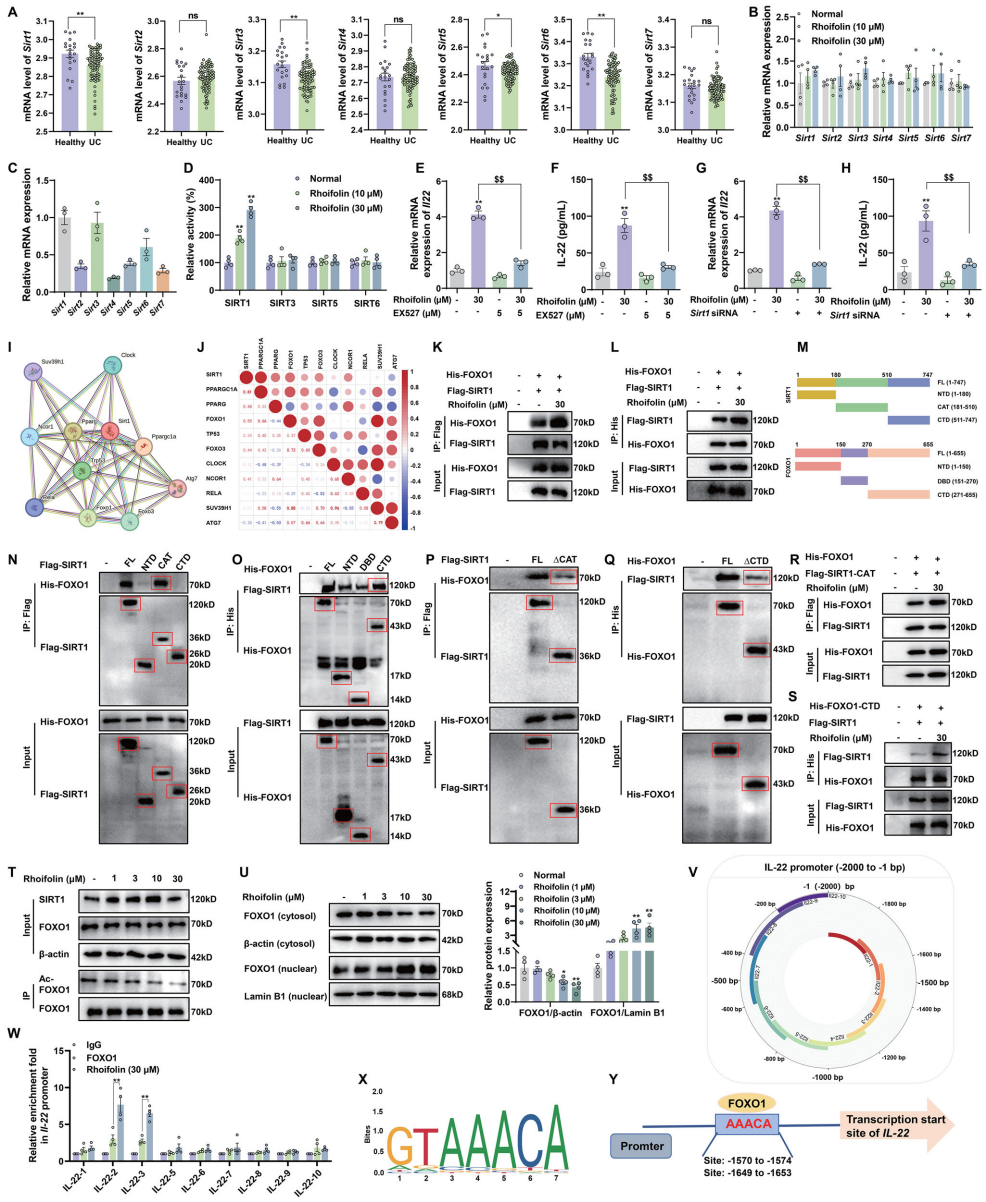
Rhoifolin potentiates ILC3s effector function via the SIRT1‐FOXO1 axis. (A) Expression profiles of SIRT family members in samples from healthy controls and colitis patients based on public datasets. (B, C) Basal and Rhoifolin‐regulated mRNA expression of *Sirt1‐7* in ILC3s. (D) Activities of SIRT1, SIRT3, SIRT5 and SIRT6 in Rhoifolin‐treated ILC3s. (E–H) ILC3s were treated with Rhoifolin in the presence of EX527 or transfected with *Sirt1* siRNA, *Il22* mRNA expression and IL‐22 production were detected by qPCR and ELISA. (I, J) STRING‐based protein‐protein interaction network and correlation analysis of SIRT1‐associated proteins. (K, L) HEK‐293T cells were transiently transfected with Flag‐SIRT1 and truncated His‐FOXO1 constructs, followed by culture with Rhoifolin (30 µM) for 24 h, the interaction between SIRT1 and FOXO1 was detected by co‐immunoprecipitation. (M) Schematic diagram showing truncated Flag‐SIRT1 and His‐FOXO1 constructs used for mapping the interaction domains. (N, O) HEK‐293T cells were transiently transfected with either Flag‐SIRT1 constructs representing different regions together with full‐length His‐FOXO1, or full‐length Flag‐SIRT1 together with truncated His‐FOXO1 constructs representing different regions for 24 h. Co‐immunoprecipitation assays were then performed to map the interaction regions between SIRT1 and FOXO1. (P, Q) HEK‐293T cells were transfected with deletion mutants lacking the identified binding domains (SIRT1‐∆CAT and FOXO1‐∆CTD), and a Co‐IP assay was performed to assess their interaction. (R, S) HEK‐293T cells were transfected with Flag‐SIRT1‐CAT with His‐FOXO1 or His‐FOXO1‐CTD with Flag‐SIRT1, followed by treatment with Rhoifolin (30 µM) for 24 h, the interaction between SIRT1 and FOXO1 was detected by co‐immunoprecipitation. (T, U) ILC3s were treated with Rhoifolin (1, 3, 10, 30 µM) for 24 h, acetylation and nuclear translocation of FOXO1 was determined by western blotting assay. (V) Schematic diagram of CUT&Tag‐qPCR primer design across the 2000‐bp upstream region of the *Il22* promoter. (W) ILC3s were treated with Rhoifolin (30 µM) for 24 h, the CUT&Tag‐qPCR was performed to evaluate FOXO1 binding at the *Il22* promoter. (X, Y) JASPAR‐based prediction and localization of FOXO1 binding motifs within the *Il22* promoter. Data were presented as mean ± S.E.M. from three independent in vitro experiments (n = 3). ^*^ *P* < 0.05, ^**^ *P* < 0.01* vs*. Healthy controls or Normal group.

To identify potential downstream effectors of SIRT1, STRING analysis predicted ten putative interacting proteins, including PPARGC1A, PPARG, TP53, FOXO3, FOXO1, CLOCK, RELA, NCOR1, ATG7, and SUV39H1 (Figure 7I). Correlation analysis and machine‐learning‐based prioritization using random forest and support vector machine models further narrowed the candidates to PPARGC1A, FOXO1, FOXO3, and PPARG (Figure 7J; Figure S7A–C). Among these candidates, Mendelian randomization analysis highlighted FOXO1 as the strongest genetically associated factor with UC susceptibility (Tables S4–S8; Figure S7D–I and Figure S8A–L). These integrated analyses nominated FOXO1 as a potential downstream effector of SIRT1.

We then examined whether Rhoifolin modulates the interaction between SIRT1 and FOXO1. Co‐immunoprecipitation assays demonstrated that Rhoifolin markedly enhanced their association (Figure 7K,L). Domain mapping using Flag‐tagged SIRT1 fragments (NTD, 1–180 aa; CAT, 180–510 aa; CTD, 511–747 aa) and His‐tagged FOXO1 fragments (NTD, 1–150 aa; DBD, 151–270 aa; CTD, 271–655 aa) demonstrated that SIRT1 primarily interacts with FOXO1 via its CAT domain, whereas FOXO1 binds SIRT1 predominantly via its CTD domain (Figure 7M–O). Deletion of these domains (SIRT1‐∆CAT or FOXO1‐∆CTD) abolished the interaction, confirming structural specificity (Figure 7P,Q). Importantly, Rhoifolin treatment further strengthened this domain‐dependent interaction (Figure 7R,S).

Consistent with the role of SIRT1 as an NAD+‐dependent deacetylase, Rhoifolin significantly reduced FOXO1 acetylation and promoted its nuclear translocation (Figure 7T,U). Given that FOXO1 functions as a transcription factor, we hypothesized that it directly regulated *Il22* transcription. Using CUT & Tag analysis combined with promoter‐targeted primers spanning the IL‐22 promoter region (ten primer pairs, Figure 7V and Table S9), Rhoifolin was found to selectively enhance FOXO1 binding at two promoter regions approximately −1800 to −1501 and −1600 to −1301 bp upstream of the transcription start site (Figure 7W). Analysis using the JASPAR database identified the FOXO1 consensus motif “AAACA” (Figure 7X). Integrating this prediction with the previously identified active promoter regions, two specific binding sites were pinpointed at −1570 to −1574 and −1649 to −1653 bp relative to the transcription start site (Figure 7Y). Together, these findings indicate that Rhoifolin facilitates SIRT1‐mediated deacetylation and nuclear localization of FOXO1, thereby promoting its direct binding to the *Il22* promoter and enhancing its transcription.

### 3.7 NMNAT1 is Indispensable for the Protective Effect of Rhoifolin in Colitis

To ultimately validate the importance of NMNAT1 in Rhoifolin‐mediated amelioration of colitis, we employed an AAV9‐mediated rectal delivery system to achieve targeted knockdown of NMNAT1 in the colon. Strikingly, depletion of NMNAT1 markedly attenuated the therapeutic benefits of Rhoifolin in DSS‐induced colitis. Specifically, the improvements in body weight loss, DAI scores, colon length, histopathological damage, neutrophil infiltration in colonic lamina propria, colonic MPO activity, and pro‐inflammatory cytokine *Il1*, *Il6*, *Tnf* mRNA expression were largely abolished (Figure 8A–H). These findings indicate that NMNAT1 is required for the anti‐colitis efficacy of Rhoifolin.

**FIGURE 8:**
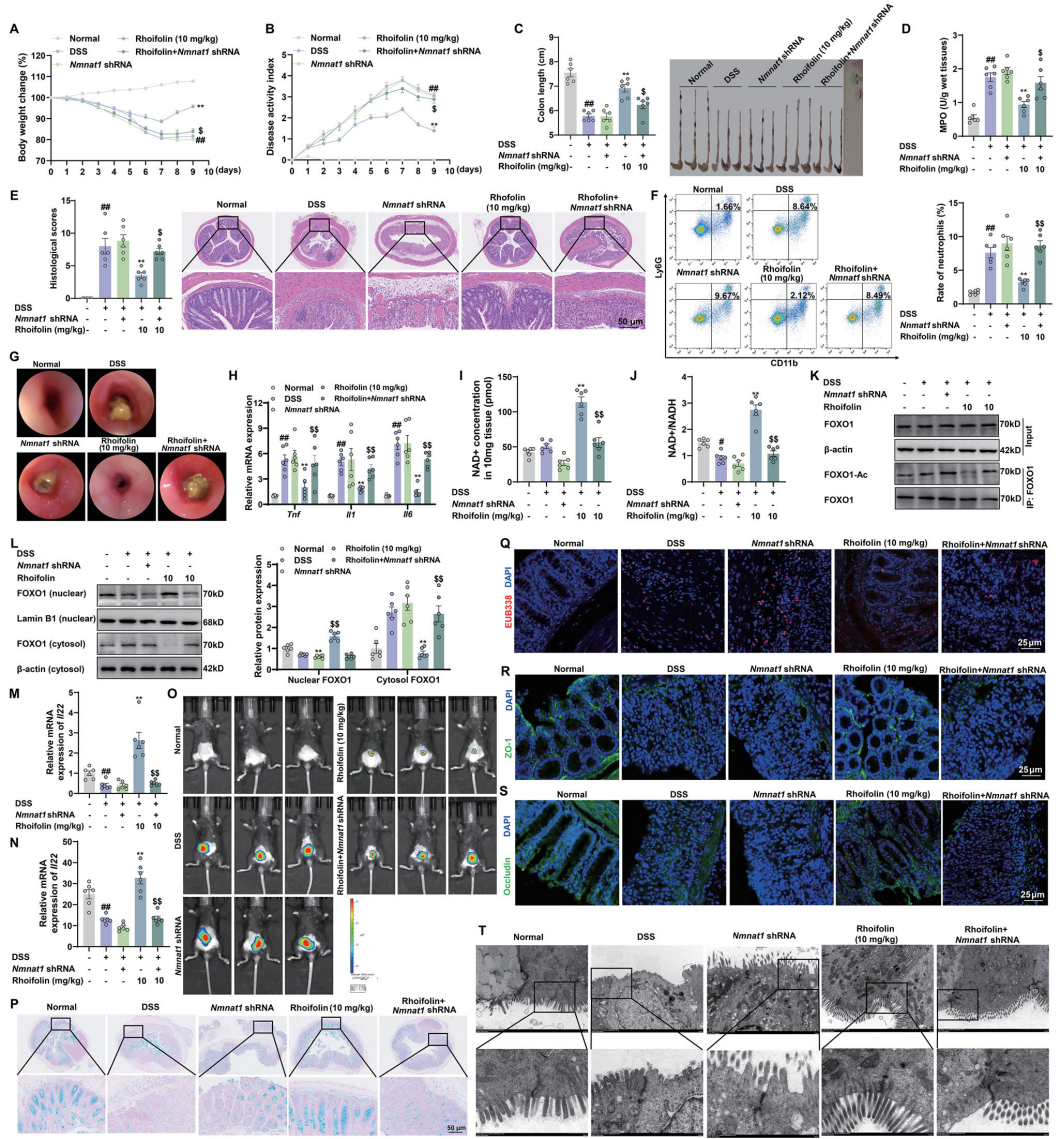
NMNAT1 is required for Rhoifolin‐mediated activation of the NAD+‐FOXO1‐IL‐22 axis and protection against DSS‐induced colitis. C57BL/6 mice were rectally administered AAV9‐*Nmnat1* shRNA 30 days prior to DSS induction, followed by daily Rhoifolin treatment throughout the experiment. (A) Body weight change. (B) DAI scores. (C) Colon length. (D) Colonic MPO activity. (E) Histological analysis of colons. (F) Neutrophil infiltration in colonic lamina propria. (G) Representative endoscopic images of colons. (H) The mRNA expression of *Il1*, *Il6*, *Tnf* and *Il22* in colons. (I, J) Colonic NAD+ levels and NAD^+^/NADH ratio. (K, L) FOXO1 acetylation and nuclear accumulation in colons were determined by western blotting assay. (M, N) Colonic IL‐22 levels. (O) Distribution and fluorescence intensity of FD4 in the abdominal region. (P) Representative Alcian blue‐stained colon sections. (Q) bacterial translocation detected by FISH using the EUB338 probe. (R, S) The representative immunofluorescence images of ZO‐1 and Occludin. (T) Representative transmission electron microscopy images of colons. Data were presented as mean ± S.E.M. of six mice in each group (n = 6). ^##^ *P* < 0.01 vs. Normal group; ^**^ *P* < 0.01* vs*. DSS group. ^$^ *p* < 0.05, ^$$^ *p* < 0.01 vs. Rhoifolin group.

We next examined whether *Nmnat1* knockdown affected the downstream NAD+‐FOXO1‐IL‐22 signaling events induced by Rhoifolin in vivo. As expected, Rhoifolin increased colonic NAD+ levels and the NAD+/NADH ratio in DSS‐challenged mice, whereas these effects were markedly reduced by *Nmnat1* knockdown (Figure 8I,J). Consistently, Rhoifolin‐induced FOXO1 deacetylation and nuclear accumulation were substantially reversed after *Nmnat1* knockdown (Figure 8K,L). In parallel, the increases in colonic IL‐22 levels were also impaired by *Nmnat1* knockdown (Figure 8M,N). Finally, *Nmnat1* knockdown compromised the barrier‐protective effects of Rhoifolin. Compared with control mice, *Nmnat1*‐deficient mice exhibited increased intestinal permeability, reduced goblet cells, enhanced bacterial encroachment, decreased ZO‐1 and Occludin expression, and disrupted epithelial ultrastructure after DSS challenge despite Rhoifolin treatment (Figure 8O–T). Collectively, these findings underscore that NMNAT1 is essential for Rhoifolin‐mediated activation of NAD+‐FOXO1‐IL‐22 axis, suppression of colonic inflammation, and preservation of epithelial barrier integrity.

## 4 Discussion

ILC3s represent a pivotal subset of the innate immune system and play indispensable roles in maintaining mucosal immune homeostasis and defending against invading pathogens at barrier surfaces, particularly within the intestine [21]. Their differentiation and maintenance are tightly regulated by cytokines including IL‐7 and IL‐15 [22]. The hallmark feature of ILC3s‐mediated immunity is the secretion of IL‐22, which reinforces epithelial barrier integrity and protects against microbial invasion [5, 6, 7]. ILC3s also response to inflammatory cues through chemokine receptors such as CCR6, α4β7 and CCR9 that guide their homing to mucosal tissues [23, 24]. Given these functions, therapeutic strategies for UC have traditionally focused on enhancing ILC3s function, improving their development, promoting their expansion, or facilitating their recruitment to inflamed intestinal sites. In the present study, Rhoifolin did not markedly affect ILC3s expansion, survival, development or migratory behavior. Instead, it selectively enhanced IL‐22 production by pre‐existing ILC3s. This finding suggests that improving the functional competence of resident ILC3s may represent a more precise strategy for restoring mucosal protection than broadly increasing ILC3s abundance or enforcing tissue recruitment. Indeed, sustained or excessive activation of homing receptors such as α4β7 or CCR9 is difficult to achieve in a stable manner and may decouple cellular migration from effector functionality, thereby limiting protective outcomes. While clinical success of vedolizumab, an antibody targeting α4β7 integrin, has validated the importance of controlling lymphocyte trafficking in UC, it also underscores the complexity and potential limitations associated with excessive modulation of immune cell migration [25, 26]. Furthermore, indiscriminate expansion of ILC3s carries the risks of disrupting immune homeostasis, potentially promoting nonspecific inflammation, and even increasing the likelihood of pathogenic IL‐17A secretion [27]. Notably, Rhoifolin enhanced IL‐22 production without substantially reshaping the intestinal ILCs compartment or altering *Il17a* expression in ILC3s, supporting pharmacological fine‐tuning of ILC3s effector function as a potential for mucosal repair in UC.

The nicotinamide salvage pathway is a central metabolic route responsible for maintaining intracellular NAD+ homeostasis. Accumulating evidence indicates that NAD+ availability profoundly influences immune cell differentiation and function. For example, nicotinamide enhances Treg function by promoting Foxp3 acetylation and stability [28]; whereas elevated intracellular NAD+ suppresses Th17 response through arginine metabolism and redox regulation [29]. However, the role of NAD+ metabolism in ILC3s biology remains poorly understood. Our study addresses this gap by demonstrating that disruption of NAD+ biosynthesis markedly impairs ILC3s effector function, whereas supplementation with either nicotinamide or NAD+ robustly enhances ILC3s activity. Importantly, this functional enhancement improves epithelial barrier integrity, establishing a direct link between NAD+ availability and ILC3s‐mediated mucosal protection. Consistently, dysregulated nicotinamide salvage pathway is also tightly linked to the pathogenesis of UC. Loss of NAD+ in intestinal epithelial cells induces mitochondrial dysfunction and intestinal inflammation [30], whereas supplementation with NAD+ precursors effectively alleviates colitis severity in mice [31]; macrophage‐specific deletion of NAMPT significantly reduces NAD+ generation and increases susceptibility to DSS‐induced colitis [32]. Together, these evidences strongly support the concept that NAD+ metabolism represents a promising therapeutic strategy against UC. Here, Rhoifolin exerts its anti‐UC effects primarily by activating nicotinamide salvage pathway, thereby elevating NAD+ levels and functionally enhancing ILC3s. We further explored the specific mechanism by which Rhoifolin elevated NAD+ levels.

NMNAT enzymes catalyze the conversion of nicotinamide mononucleotide (NMN) into NAD+ and comprise three mammalian isoforms with distinct subcellular distributions and biological functions. NMNAT1 is predominantly nuclear and maintains nuclear NAD+ pools that support transcriptional and epigenetic regulation, whereas NMNAT2 is mainly cytoplasmic and enriched in neurons, and NMNAT3 is predominantly mitochondrial and involved in mitochondrial NAD+ homeostasis. Given that IL‐22 expression in ILC3s is tightly regulated at the transcriptional level, NMNAT1 represents a particularly relevant link between NAD+ metabolism and *Il22* transcription. Here, Rhoifolin directly targeted NMNAT1 and promoted the nicotinamide salvage pathway, thereby increasing nuclear NAD+ availability and coupling metabolic activation to SIRT1‐FOXO1‐dependent *Il22* transcription, an effect further validated in colitis mice. Nevertheless, the AAV9‐shRNA strategy used for *in vivo Nmnat1* knockdown was not strictly ILC3s‐specific. Because NMNAT1 is not restricted to ILC3s, contributions from other intestinal cell populations, including epithelial cells, macrophages, dendritic cells and T cells, cannot be fully excluded. Thus, future studies using ILC3s‐targeted genetic deletion or adoptive transfer of *Nmnat1*‐deficient ILC3s will be needed to more precisely define the ILC3s‐intrinsic role of NMNAT1 in Rhoifolin‐mediated mucosal protection.

We further investigated how elevated NAD+ levels translate into enhanced ILC3s effector function. NAD+ is an essential cofactor for the SIRT family of deacetylases. Among SIRT members implicated in UC pathogenesis, Rhoifolin selectively enhanced SIRT1 enzymatic activity. SIRT1 is known to suppress inflammatory response through deacetylation of NF‐κB and modulates Treg activation via Notch1 signaling [33, 34]. Its protective role in colitis has also been well documented: reduced SIRT1 expression exacerbates inflammation, oxidative stress, and mucosal barrier disruption [35, 36], whereas pharmacological activation of SIRT1 alleviates intestinal inflammation and restores barrier function [37, 38]. Consistent with these observations, our findings suggest that Rhoifolin enhances ILC3s effector function through potentiation of SIRT1 activity. A thought‐provoking question arises: how does this enhanced activity regulate ILC3s function? Previous evidence has shown that SIRT1 can stabilize β‐TrCP1, suppress Snail1 expression, and promote tight junction proteins (E‐cadherin, Occludin, Claudin‐1) expression, ultimately restoring barrier integrity and suppressing inflammation [39]. However, our in vitro experiments using Caco‐2 cells suggest that Rhoifolin does not directly regulate these junctional proteins. This finding points to a more indirect regulatory mechanism, whereby SIRT1 may act on downstream mediators that bridge metabolic activation and ILC3s‐mediated epithelial barrier repair.

To identify such mediators, we performed a stepwise integrative screening strategy incorporating protein–protein interaction analysis, correlation assessment, machine learning‐based prioritization, and Mendelian randomization, which converged on FOXO1 as the top candidate. Notably, this unbiased identification is well aligned with the established roles of FOXO1 in metabolic regulation, cell apoptosis, and immune responses [40, 41]. FOXO1 activity is tightly controlled by multiple post‐translational modifications that regulate its subcellular localization, stability, and transcriptional activity [42, 43, 44]. Among these modifications, acetylation is particularly important because it can impair FOXO1 DNA‐binding capacity, alter cofactor interactions, and thereby shape immune‐cell fate and function. Ren H et al. demonstrated that FOXO1 negatively correlates with the Th17‐promoting gene IRF5, thereby dampening Th17 differentiation to ameliorate hepatocellular carcinoma recurrence after hepatic ischemia‐reperfusion injury [45]. Similarly, enhanced FOXO1 nuclear translocation promoted the transcription of Foxp3, thus enhancing Treg differentiation to alleviate ischemia‐reperfusion injury [46]. In line with this mechanism, we found that Rhoifolin promoted FOXO1 deacetylation and nuclear accumulation in ILC3s, accompanied by increased FOXO1 binding to the *Il22* promoter. FOXO1 has previously been reported to antagonize the Th17 program through its interaction with RORγt, thereby limiting RORγt‐dependent genes including *Il17a* and *Il23r* [47, 48]. In our study, however, Rhoifolin‐induced FOXO1 activation did not markedly alter *Il17a* expression in ILC3s. Instead, Rhoifolin selectively enhanced IL‐22 production and increased FOXO1 occupancy at the *Il22* promoter. These differences may reflect the cell‐type‐specific regulatory context in which FOXO1 operates. Although Th17 cells and ILC3s both express RORγt, they differ in developmental origin, chromatin accessibility, and cytokine‐regulatory networks. Therefore, in intestinal ILC3s, Rhoifolin‐induced FOXO1 activation may preferentially support *Il22* transcription rather than recapitulating the IL‐17‐regulatory effects described in Th17 cells.

Despite these mechanistic insights, several limitations should be considered before Rhoifolin can be further developed for UC therapy. First, although Rhoifolin showed protective effects in experimental colitis models, these models do not fully recapitulate the clinical heterogeneity, relapsing course, genetic complexity and treatment history. Its efficacy therefore requires validation in clinically relevant models. Second, as a flavonoid glycoside, Rhoifolin may face pharmacokinetic challenges, including limited oral absorption, variable intestinal exposure, rapid clearance and uncertain tissue distribution. Future studies should establish a clear pharmacokinetic/pharmacodynamic relationship linking intestinal Rhoifolin exposure, NMNAT1 engagement, SIRT1 activation and IL‐22 induction. Third, because NMNAT1 is not ILC3s‐specific, contributions of other intestinal epithelial or immune cells cannot be fully excluded, and cell type‐specific genetic approaches are needed to clarify the contribution of NMNAT1 in ILC3s. Finally, although IL‐22 is generally barrier‐protective during mucosal injury, prolonged or excessive IL‐22 signaling may promote epithelial proliferation under chronic inflammatory conditions. Thus, the optimal dose and long‐term safety of Rhoifolin require further systematic evaluation.

These findings also highlight both the promise and challenges of developing single‐component traditional Chinese medicine‐derived compounds for UC therapy. Compared with crude extracts or herbal formulas, single compounds offer advantages in chemical structure definition, quality control, dosing standardization, target identification, and more straightforward pharmacokinetic and toxicological evaluation. These features make them suitable for mechanism‐based drug development. Together, our study provides a mechanistic basis for developing Rhoifolin as an ILC3s‐directed immunometabolic modulator, while underscoring the need for rigorous translational evaluation before clinical application.

## 5 Conclusion

In summary, our study identifies Rhoifolin as a previously unrecognized small‐molecule enhancer of ILC3s effector function that effectively suppresses intestinal inflammation. Mechanistically, Rhoifolin directly engages NMNAT1 and enhances its enzymatic activity, thereby promoting the nicotinamide salvage pathway and increasing intracellular NAD+ availability. Elevated NAD+ subsequently potentiates SIRT1 activity, leading to deacetylation and nuclear translocation of FOXO1. Nuclear FOXO1 subsequently binds to the *Il22* promoter and activates its transcription, thereby enhancing IL‐22 production by ILC3s and reinforcing intestinal epithelial barrier integrity. Collectively, our findings unveil a previously unrecognized NMNAT1‐NAD+‐SIRT1‐FOXO1‐IL‐22 axis through which Rhoifolin potentiates ILC3s‐mediated mucosal protection, providing mechanistic insight and a promising therapeutic avenue for UC.

## Author Contributions

**Lihong Hu**, **Qi Lv** and **Caijuan Zheng** conceived of the study and designed the experiments. **Hongqiong Yang**, **Yishu Zhang**, **Sijia Feng** and **Xiaoqing Ma** interpreted the experiments, prepared the images for publication and drafted the manuscript. **Jialin Gao** and **An Pan** aided in experimental design, provided technical support and proofread the manuscript. **Dapeng Wu** and **Kang Ding** generated the DSS‐and TNBS‐induced colitis models. **Jiayi Feng** and **Junwei Wang** performed the phenotypic analysis of ILC subsets.

## Funding

This work was supported by the Jiangsu Province Traditional Chinese Medicine Science and Technology Development Plan Project (No. QN202409); Medical Scientific Research Project of Jiangsu Commission of Health (No. ZQ2024003); the key research projects of Jiangsu Higher Education (No. 25KJA360005); Leading Discipline Phase IV Traditional Chinese Medicine‐Anorectal Surgery Fund (035062005006‐42); the open foundation of Key Laboratory of Tropical Medicinal Resource Chemistry of Ministry of Education (No. RDZH2024003).

## Ethics Approval

All animal experiments in the present study were conducted in accordance with the Guide for the Care and Use of Laboratory Animals, and the protocol was approved by the Animal Ethics Committee of Nanjing University of Chinese Medicine.

## Consent for Publication

All authors read and approved the final manuscript.

## Conflicts of Interest

The authors declare no conflicts of interest.

## Supporting information




**Supporting File 1**: advs76935‐sup‐0001‐SuppMat.docx.

**Supporting File 2**: advs76935‐sup‐0002‐TableS1‐S9.docx.

## Data Availability

All data generated or analyzed during this study are included in this article and its supplementary files.
