# Supplementary material for: Rhoifolin Alleviates Ulcerative Colitis by Targeting NMNAT1 and Activating SIRT1‐FOXO1 Signaling to Enhance ILC3s Effector Function
Source: Adv Sci (Weinh). 2026 Aug 7;13(60):e76935. doi: 10.1002/advs.76935 (PMC13449538; doi:10.1002/advs.76935)
Supplement: Supplementary file 1 [file ADVS-13-e76935-s001.docx]

**Supplementary Tables**

Supplementary Table 1. Sequences of siRNA for gene knockdown

| siRNA | Sense (5’-3’) | Antiense (5’-3’) |
| --- | --- | --- |
| *Sirt1* siRNA | GGGAUCAAGAGGUUGUUAA(dT)(dT) | UUAACAACCUCUUGAUCCC(dT)(dT) |
| *Nmnat1* siRNA | GGACUAUAUGCAUGCUACA(dT)(dT) | UGUAGCAUGCAUAUAGUCC(dT)(dT) |
| *Nmnat2* siRNA | GCACAAGACUGGAAGAUUU(dT)(dT) | AAAUCUUCCAGUCUUGUGC(dT)(dT) |
| *Nmnat3* siRNA | GGAAAGACACGCACAUCCA(dT)(dT) | UGGAUGUGCGUGUCUUUCC(dT)(dT) |

Supplementary Table 2. Differentially altered metabolites identified in Rhoifolin-treated ILC3s

| Alignment ID | Metabolite name | Category1 | Category2 | HMDB ID | log2fc | p.value | fdr | VIP | Regulation |
| --- | --- | --- | --- | --- | --- | --- | --- | --- | --- |
| Meta_109 | N-Acetyltaurine | Amino Acid | Methionine, Cysteine, SAM and Taurine Metabolism | HMDB0240253 | -0.166 | 0.006219 | 0.006219 | 2.118359 | down |
| Meta_383 | LPC 20:2_sn1 | Lipid | Glycerophospholipid (lysophosphatidylcholine) | HMDB0010392 | -0.58733 | 0.045976 | 0.045976 | 1.915039 | down |
| Meta_54 | L-Pipecolic Acid | Amino Acid | Lysine Metabolism | HMDB0000716 | -0.302 | 0.027085 | 0.027085 | 2.006275 | down |
| Meta_236 | 2-Methylcytidine | Nucleotide | Pyrimidine Metabolism, Cytidine containing | HMDB0242132 | 0.705667 | 0.022305 | 0.022305 | 2.004025 | up |
| Meta_310 | Fructose 1,6-Bisphosphate | Carbohydrate | Glycolysis, Gluconeogenesis, and Pyruvate Metabolism | HMDB0001058 | 0.388667 | 0.011887 | 0.011887 | 2.078534 | up |
| Meta_370 | LPE 20:5_sn2 | Lipid | Glycerophospholipid (lysophosphatidylethanolamine) | HMDB0011519 | 0.453333 | 0.028322 | 0.028322 | 1.967704 | up |
| Meta_50 | Citraconic Acid | Lipid | Fatty Acid, Dicarboxylate | HMDB0000634 | 0.304667 | 0.012126 | 0.012126 | 2.100336 | up |
| Meta_7 | Dimethyl Sulfoxide | Xenobiotics | Chemical | HMDB0002151 | 8.225333 | 0.000567 | 0.000567 | 2.267412 | up |
| Meta_70 | 1-Methylnicotinamide | Cofactors and Vitamins | Nicotinate and Nicotinamide Metabolism | HMDB0000699 | 1.423 | 0.016632 | 0.016632 | 2.146569 | up |

Supplementary Table 3. Binding free energy decomposition of the Rhoifolin-NMNAT1 complex

| Contribution | kcal·mol-1) |
| --- | --- |
| ∆Evdwa | -37.80±2.36 |
| ∆Eeleb | -52.70±2.22 |
| ∆EGBc | 34.58±1.80 |
| ∆EGAd | -42.62±2.46 |
| ∆Ebinde | -99.36±3.20 |

Supplementary Table 4. Instrumental variables of genes (PPARGC1A)

| Gene | ENSEMBL ID | SNP | effect_allele.exposure | other_allele.exposure | beta.exposure | eaf.exposure | pval.exposure | se.exposure | pos.exposure | chr.exposure | samplesize.exposure | F |
| --- | --- | --- | --- | --- | --- | --- | --- | --- | --- | --- | --- | --- |
| PPARGC1A | ENSG00000109819 | rs115179278 | T | C | -0.388111 | 0.0286023 | 9.72971e-28 | 0.0355566 | 23879277 | 4 | 29636 | 119.143801116855 |
| PPARGC1A | ENSG00000109819 | rs61196244 | C | T | 0.169074 | 0.041423 | 1.45322e-08 | 0.0298343 | 23801790 | 4 | 26283 | 32.1160370249473 |
| PPARGC1A | ENSG00000109819 | rs74893741 | G | A | 0.710265 | 0.197313 | 1e-200 | 0.0137084 | 23867367 | 4 | 31239 | 2684.52400639179 |
| PPARGC1A | ENSG00000109819 | rs77371311 | A | C | 0.249499 | 0.0353734 | 8.49376e-15 | 0.0321516 | 24030320 | 4 | 30532 | 60.2188473372297 |

Supplementary Table 5. Instrumental variables of genes (PPARG)

| Gene | ENSEMBL ID | SNP | effect_allele.exposure | other_allele.exposure | beta.exposure | eaf.exposure | pval.exposure | se.exposure | pos.exposure | chr.exposure | samplesize.exposure | F |
| --- | --- | --- | --- | --- | --- | --- | --- | --- | --- | --- | --- | --- |
| PPARG | ENSG00000132170 | rs111536786 | A | G | -0.227925 | 0.0223079 | 1.4962e-08 | 0.0402552 | 12279153 | 3 | 17774 | 32.0582602031857 |
| PPARG | ENSG00000132170 | rs113462615 | C | T | -0.248789 | 0.0425357 | 2.73842e-17 | 0.0294177 | 12597892 | 3 | 29384 | 71.5228677562067 |
| PPARG | ENSG00000132170 | rs114391178 | G | A | -0.252972 | 0.0188741 | 6.99697e-09 | 0.0436851 | 12576358 | 3 | 24864 | 33.5334502511731 |
| PPARG | ENSG00000132170 | rs115110658 | A | G | -0.245418 | 0.0179186 | 4.35542e-08 | 0.0448186 | 12496557 | 3 | 27175 | 29.9844614190814 |
| PPARG | ENSG00000132170 | rs115718559 | A | G | 0.203634 | 0.0328865 | 9.98803e-10 | 0.0333291 | 12785829 | 3 | 26558 | 37.3296064783563 |
| PPARG | ENSG00000132170 | rs11706443 | G | T | -0.116646 | 0.119235 | 2.01229e-10 | 0.0183396 | 11981472 | 3 | 31355 | 40.4538614745315 |
| PPARG | ENSG00000132170 | rs11718084 | C | T | 0.327316 | 0.0541268 | 6.40324e-36 | 0.0261593 | 12592368 | 3 | 26617 | 156.560512190747 |
| PPARG | ENSG00000132170 | rs1175544 | T | C | -0.0816168 | 0.311927 | 1.98879e-10 | 0.0128286 | 12467044 | 3 | 31017 | 40.4762806434666 |
| PPARG | ENSG00000132170 | rs1185783 | G | A | -0.212137 | 0.90222 | 2.19381e-26 | 0.0199587 | 12490986 | 3 | 31563 | 112.971356891176 |
| PPARG | ENSG00000132170 | rs146058284 | C | T | -0.23106 | 0.0240681 | 2.56277e-09 | 0.0387854 | 12143636 | 3 | 20255 | 35.4905711501755 |
| PPARG | ENSG00000132170 | rs149549882 | A | C | -0.251645 | 0.0242105 | 7.59626e-11 | 0.0386646 | 12572329 | 3 | 24075 | 42.3593774022861 |
| PPARG | ENSG00000132170 | rs150535373 | A | G | -0.291609 | 0.0190316 | 2.00863e-11 | 0.0434899 | 12410328 | 3 | 21727 | 44.9598681626627 |
| PPARG | ENSG00000132170 | rs150637589 | A | G | 0.307652 | 0.0220253 | 2.92012e-14 | 0.0404699 | 12772512 | 3 | 24825 | 57.7903364515652 |
| PPARG | ENSG00000132170 | rs150919536 | T | C | -0.228527 | 0.0285328 | 1.53349e-10 | 0.0356968 | 12717533 | 3 | 29100 | 40.9842002521757 |
| PPARG | ENSG00000132170 | rs1619599 | A | G | -0.36722 | 0.543714 | 1e-200 | 0.0115426 | 12504290 | 3 | 30902 | 1012.15112226116 |
| PPARG | ENSG00000132170 | rs1661596 | C | T | 0.130794 | 0.744861 | 7.14332e-22 | 0.0136082 | 12254130 | 3 | 31684 | 92.3792169327248 |
| PPARG | ENSG00000132170 | rs17279604 | C | G | -0.250363 | 0.0672115 | 3.91652e-26 | 0.0236765 | 12523604 | 3 | 31566 | 111.816343969042 |
| PPARG | ENSG00000132170 | rs2442803 | A | G | -0.296138 | 0.477658 | 1.69434e-142 | 0.0116518 | 12610074 | 3 | 31684 | 645.954956622706 |
| PPARG | ENSG00000132170 | rs2618401 | T | C | 0.220882 | 0.070795 | 1.30707e-21 | 0.0231307 | 12042001 | 3 | 31567 | 91.1891358486755 |
| PPARG | ENSG00000132170 | rs2972165 | C | T | -0.235417 | 0.853349 | 4.3381e-45 | 0.0167072 | 12337875 | 3 | 31355 | 198.549271997117 |
| PPARG | ENSG00000132170 | rs299631 | C | T | -0.255274 | 0.954043 | 2.1252e-19 | 0.0283427 | 12570050 | 3 | 29141 | 81.1205202636853 |
| PPARG | ENSG00000132170 | rs3105364 | G | A | 0.308208 | 0.0168492 | 2.46547e-11 | 0.0461701 | 12468690 | 3 | 22791 | 44.5621549278201 |
| PPARG | ENSG00000132170 | rs310749 | T | G | 0.136681 | 0.487917 | 9.01571e-31 | 0.0118513 | 12273768 | 3 | 31682 | 133.010005504413 |
| PPARG | ENSG00000132170 | rs3112394 | T | C | 0.345829 | 0.0170064 | 5.1606e-14 | 0.0459402 | 12353303 | 3 | 22980 | 56.6678916195117 |
| PPARG | ENSG00000132170 | rs35240997 | G | A | -0.103789 | 0.230354 | 1.88365e-13 | 0.0141082 | 12379351 | 3 | 31569 | 54.120205852603 |
| PPARG | ENSG00000132170 | rs3773341 | A | G | 0.127483 | 0.30552 | 4.13809e-23 | 0.0128763 | 12616533 | 3 | 30746 | 98.021733069379 |
| PPARG | ENSG00000132170 | rs3864029 | A | G | -0.234156 | 0.0314997 | 5.84386e-12 | 0.0340184 | 12547160 | 3 | 27980 | 47.3786644554608 |
| PPARG | ENSG00000132170 | rs3894895 | C | T | -0.0875137 | 0.778212 | 9.54729e-10 | 0.0143071 | 12888976 | 3 | 31562 | 37.4152685078151 |
| PPARG | ENSG00000132170 | rs392394 | A | G | 0.167317 | 0.776452 | 5.59887e-32 | 0.0142161 | 12140087 | 3 | 31566 | 138.522142027648 |
| PPARG | ENSG00000132170 | rs4135292 | A | G | 0.356788 | 0.0201735 | 2.93292e-17 | 0.0422264 | 12465956 | 3 | 26291 | 71.392462745961 |
| PPARG | ENSG00000132170 | rs4684833 | T | C | 0.160675 | 0.779139 | 2.34207e-29 | 0.0142837 | 12031719 | 3 | 31569 | 126.536313287179 |
| PPARG | ENSG00000132170 | rs4684858 | T | G | 0.109258 | 0.429726 | 7.93963e-20 | 0.0119876 | 12498350 | 3 | 31564 | 83.0695784567385 |
| PPARG | ENSG00000132170 | rs60710264 | C | A | -0.142902 | 0.12247 | 3.05633e-15 | 0.0181152 | 12484928 | 3 | 31355 | 62.2286454528853 |
| PPARG | ENSG00000132170 | rs62242087 | T | C | -0.145716 | 0.0643727 | 1.78341e-09 | 0.0242207 | 12302208 | 3 | 31173 | 36.1943766221934 |
| PPARG | ENSG00000132170 | rs6442327 | G | A | -0.122691 | 0.509057 | 4.43711e-25 | 0.0118606 | 12778794 | 3 | 31684 | 107.006976553233 |
| PPARG | ENSG00000132170 | rs73022986 | T | C | -0.150913 | 0.0653861 | 3.45168e-10 | 0.0240425 | 12801056 | 3 | 25691 | 39.3998034791796 |
| PPARG | ENSG00000132170 | rs75348895 | A | G | -0.235289 | 0.0987072 | 2.15179e-32 | 0.0198558 | 12735637 | 3 | 31684 | 140.41983827146 |
| PPARG | ENSG00000132170 | rs7642826 | A | G | -0.1881 | 0.952817 | 1.92486e-11 | 0.0280257 | 12770751 | 3 | 25538 | 45.0468734524462 |
| PPARG | ENSG00000132170 | rs78537053 | G | A | -0.159269 | 0.0437539 | 4.26285e-08 | 0.0290663 | 12529085 | 3 | 29130 | 30.0249991068326 |
| PPARG | ENSG00000132170 | rs79186078 | C | T | 0.338785 | 0.0271784 | 1.63531e-20 | 0.0364917 | 12191441 | 3 | 29451 | 86.1906498044982 |
| PPARG | ENSG00000132170 | rs79310821 | A | G | 0.197711 | 0.0313757 | 6.71908e-09 | 0.0340999 | 12326565 | 3 | 30923 | 33.6167291189071 |
| PPARG | ENSG00000132170 | rs9809905 | G | T | -0.214334 | 0.393138 | 9.07194e-71 | 0.0120508 | 12500651 | 3 | 31684 | 316.337283247349 |
| PPARG | ENSG00000132170 | rs9849171 | C | G | -0.175186 | 0.352871 | 1.50453e-45 | 0.0123673 | 12622623 | 3 | 31562 | 200.654545056978 |
| PPARG | ENSG00000132170 | rs9874315 | A | C | 0.253595 | 0.456481 | 3.41979e-103 | 0.0117566 | 12727962 | 3 | 31684 | 465.283753713273 |

Supplementary Table 6. Instrumental variables of genes (FOXO1)

| Gene | ENSEMBL ID | SNP | effect_allele.exposure | other_allele.exposure | beta.exposure | eaf.exposure | pval.exposure | se.exposure | pos.exposure | chr.exposure | samplesize.exposure | F |
| --- | --- | --- | --- | --- | --- | --- | --- | --- | --- | --- | --- | --- |
| FOXO1 | ENSG00000150907 | rs11619171 | A | G | -0.146864 | 0.0215049 | 0.000342019 | 0.0410109 | 41000364 | 13 | 28651 | 12.8242538098127 |
| FOXO1 | ENSG00000150907 | rs117073308 | C | T | -0.0648927 | 0.124957 | 0.000309763 | 0.0179908 | 41275319 | 13 | 31228 | 13.0104026464383 |
| FOXO1 | ENSG00000150907 | rs139485965 | A | G | -0.152934 | 0.0166174 | 0.0010155 | 0.046541 | 41622214 | 13 | 21985 | 10.7978346912532 |
| FOXO1 | ENSG00000150907 | rs141719030 | C | T | -0.190136 | 0.0334954 | 8.67441e-09 | 0.0330401 | 41149864 | 13 | 28735 | 33.1166196497515 |
| FOXO1 | ENSG00000150907 | rs141726773 | C | T | -0.146181 | 0.022123 | 0.000301037 | 0.0404464 | 41810092 | 13 | 24331 | 13.062373928346 |
| FOXO1 | ENSG00000150907 | rs150175041 | T | G | -0.109224 | 0.0314828 | 0.00134688 | 0.0340719 | 41627824 | 13 | 25213 | 10.2764579519908 |
| FOXO1 | ENSG00000150907 | rs1810779 | C | T | -0.0589046 | 0.725464 | 9.87916e-06 | 0.0133271 | 40916518 | 13 | 25260 | 19.535615975331 |
| FOXO1 | ENSG00000150907 | rs1885267 | G | A | 0.0398064 | 0.508828 | 0.00082283 | 0.0119006 | 41122647 | 13 | 30902 | 11.1884027013742 |
| FOXO1 | ENSG00000150907 | rs2324597 | G | A | -0.0737096 | 0.293864 | 1.6233e-08 | 0.0130508 | 40918250 | 13 | 30631 | 31.8987628812016 |
| FOXO1 | ENSG00000150907 | rs2802494 | A | G | -0.073745 | 0.800483 | 7.20328e-07 | 0.0148799 | 41066672 | 13 | 31564 | 24.5620796185945 |
| FOXO1 | ENSG00000150907 | rs35504306 | G | T | 0.0532229 | 0.346392 | 2.06628e-05 | 0.0125004 | 40939453 | 13 | 30077 | 18.1279731313805 |
| FOXO1 | ENSG00000150907 | rs3751437 | G | A | -0.0458588 | 0.22696 | 0.00124397 | 0.0142039 | 41133080 | 13 | 31355 | 10.4238981746215 |
| FOXO1 | ENSG00000150907 | rs41286971 | A | G | 0.0492096 | 0.412135 | 4.66112e-05 | 0.0120846 | 41026812 | 13 | 31684 | 16.5819312459937 |
| FOXO1 | ENSG00000150907 | rs56302456 | T | G | 0.0911076 | 0.189391 | 1.90858e-09 | 0.0151707 | 41295887 | 13 | 31684 | 36.0659994915478 |
| FOXO1 | ENSG00000150907 | rs61963209 | A | G | 0.0744652 | 0.210861 | 3.25237e-07 | 0.014577 | 41502588 | 13 | 31564 | 26.095787847093 |
| FOXO1 | ENSG00000150907 | rs61963554 | T | G | 0.0924388 | 0.179308 | 2.44039e-09 | 0.0154956 | 40986282 | 13 | 30902 | 35.5870348721397 |
| FOXO1 | ENSG00000150907 | rs61964871 | T | C | 0.0600901 | 0.240787 | 1.56452e-05 | 0.013911 | 41677832 | 13 | 31559 | 18.6590338373761 |
| FOXO1 | ENSG00000150907 | rs73171104 | G | C | -0.0848613 | 0.150037 | 3.45812e-07 | 0.0166512 | 40891162 | 13 | 30902 | 25.973369060811 |
| FOXO1 | ENSG00000150907 | rs73173135 | C | T | 0.11351 | 0.036777 | 0.000329412 | 0.0316078 | 41081667 | 13 | 31355 | 12.8967330374185 |
| FOXO1 | ENSG00000150907 | rs74044567 | T | C | -0.149313 | 0.0354794 | 3.40738e-06 | 0.0321491 | 41453014 | 13 | 26059 | 21.5703704484624 |
| FOXO1 | ENSG00000150907 | rs75560776 | T | C | 0.149336 | 0.0165057 | 0.00138385 | 0.0466966 | 40371357 | 13 | 23190 | 10.2272414200434 |
| FOXO1 | ENSG00000150907 | rs77526211 | A | G | -0.251217 | 0.0432109 | 7.63133e-18 | 0.0291947 | 40974023 | 13 | 29143 | 74.0440245773017 |
| FOXO1 | ENSG00000150907 | rs78790120 | T | C | -0.0924378 | 0.04677 | 0.00103588 | 0.0281771 | 40957298 | 13 | 29781 | 10.7623376965921 |
| FOXO1 | ENSG00000150907 | rs9532534 | T | C | 0.0700724 | 0.228494 | 7.52108e-07 | 0.0141632 | 40924528 | 13 | 31684 | 24.4777338934755 |
| FOXO1 | ENSG00000150907 | rs9532563 | C | T | 0.0986507 | 0.228975 | 3.02831e-12 | 0.0141406 | 41160270 | 13 | 31567 | 48.6703722228753 |
| FOXO1 | ENSG00000150907 | rs9549200 | C | T | 0.126609 | 0.613666 | 2.548e-25 | 0.012177 | 40976249 | 13 | 31556 | 108.105688022098 |
| FOXO1 | ENSG00000150907 | rs9549297 | G | A | 0.0853595 | 0.187826 | 2.05149e-08 | 0.0152216 | 41397482 | 13 | 31565 | 31.4472819640636 |
| FOXO1 | ENSG00000150907 | rs9566607 | T | G | -0.0455101 | 0.280318 | 0.000590364 | 0.0132455 | 41495924 | 13 | 31561 | 11.805349951917 |

Supplementary Table 7. Instrumental variables of genes (FOXO3)

| Gene | ENSEMBL ID | SNP | effect_allele.exposure | other_allele.exposure | beta.exposure | eaf.exposure | pval.exposure | se.exposure | pos.exposure | chr.exposure | samplesize.exposure | F |
| --- | --- | --- | --- | --- | --- | --- | --- | --- | --- | --- | --- | --- |
| FOXO3 | ENSG00000240445 | rs11078419 | A | G | -0.154558 | 0.0825597 | 8.06678e-13 | 0.0215866 | 18627407 | 17 | 6421 | 51.2642361110977 |
| FOXO3 | ENSG00000240445 | rs112795111 | A | G | -0.183975 | 0.0602065 | 1.74502e-13 | 0.0249731 | 18789963 | 17 | 5258 | 54.2716106717974 |
| FOXO3 | ENSG00000240445 | rs117607818 | T | C | 0.177781 | 0.0377682 | 1.1925e-08 | 0.0311847 | 18828406 | 17 | 5258 | 32.5003133754391 |
| FOXO3 | ENSG00000240445 | rs12603319 | T | C | -0.105813 | 0.203921 | 7.15649e-13 | 0.014745 | 18661327 | 17 | 6421 | 51.4977792772142 |
| FOXO3 | ENSG00000240445 | rs140651876 | T | C | 0.172365 | 0.042068 | 5.86368e-09 | 0.0296129 | 18608936 | 17 | 3906 | 33.8794450015554 |
| FOXO3 | ENSG00000240445 | rs1625912 | C | T | 0.182012 | 0.381661 | 1.09245e-50 | 0.0121554 | 18838080 | 17 | 6406 | 224.213384312339 |
| FOXO3 | ENSG00000240445 | rs163367 | G | A | 0.13924 | 0.780637 | 2.63876e-22 | 0.0143348 | 18618644 | 17 | 6083 | 94.3506188746295 |
| FOXO3 | ENSG00000240445 | rs2270139 | G | A | 0.183546 | 0.422811 | 2.97098e-53 | 0.0119485 | 18702094 | 17 | 6083 | 235.973412453537 |
| FOXO3 | ENSG00000240445 | rs2746029 | G | C | -0.0862098 | 0.399713 | 1.17733e-12 | 0.0121287 | 18142740 | 17 | 6416 | 50.5224923796931 |
| FOXO3 | ENSG00000240445 | rs34824853 | A | G | 0.144882 | 0.0622351 | 3.91372e-09 | 0.0246063 | 18219742 | 17 | 6421 | 34.66859454 |
| FOXO3 | ENSG00000240445 | rs4441330 | C | T | -0.118098 | 0.530326 | 2.8622e-23 | 0.011884 | 18562720 | 17 | 6421 | 98.7551605665039 |
| FOXO3 | ENSG00000240445 | rs4449005 | C | T | 0.244182 | 0.578089 | 5.86408e-94 | 0.0118748 | 18579709 | 17 | 6083 | 422.838657706978 |
| FOXO3 | ENSG00000240445 | rs638318 | G | A | 0.0816392 | 0.630769 | 3.3566e-11 | 0.0123136 | 18258150 | 17 | 2337 | 43.9569344594662 |
| FOXO3 | ENSG00000240445 | rs7215146 | C | A | 0.125129 | 0.924338 | 2.60351e-08 | 0.0224809 | 18822626 | 17 | 5262 | 30.9805098018269 |
| FOXO3 | ENSG00000240445 | rs7215146 | C | A | 0.125129 | 0.924338 | 2.60351e-08 | 0.0224809 | 18822626 | 17 | 5262 | 30.9805098018269 |

Supplementary Table 8. MR results of PPARGC1A、PPARG、FOXO1、FOXO3 and UC

| Trails | exposure | method | nsnp | pval | or |
| --- | --- | --- | --- | --- | --- |
| Ulcerative colitis | PPARGC1A | MR Egger | 4 | 0.310068544435347 | 0.9991(0.9978-1.0004) |
|  |  | Weighted median | 4 | 0.308056500271603 | 0.9997(0.9992-1.0002) |
|  |  | Inverse variance weighted | 4 | 0.371780871805141 | 0.9998(0.9992-1.0003) |
|  |  | Simple mode | 4 | 0.289719757587968 | 1.0011(0.9994-1.0029) |
|  |  | Weighted mode | 4 | 0.232811074669511 | 0.9996(0.9991-1.0001) |
|  | PPARG | MR Egger | 44 | 0.268802067446976 | 1.0005（0.9996-1.0013） |
|  |  | Weighted median | 44 | 0.837300765980798 | 1.0001（0.9995-1.0006） |
|  |  | Inverse variance weighted | 44 | 0.265144396190001 | 0.9998（0.9995-1.0001） |
|  |  | Simple mode | 44 | 0.183930014833705 | 0.9993（0.9984-1.0003） |
|  |  | Weighted mode | 44 | 0.843249569884675 | 0.9999（0.9994-1.0005） |
|  | FOXO1 | MR Egger | 28 | 0.823680925286937 | 1.0003(0.9979-1.0027) |
|  |  | Weighted median | 28 | 0.498355851054702 | 1.0005(0.9991-1.0018) |
|  |  | Inverse variance weighted | 28 | 0.0434884253276442 | 1.0010(1.0000-1.0019) |
|  |  | Simple mode | 28 | 0.59913795875499 | 1.0007(0.9982-1.0031) |
|  |  | Weighted mode | 28 | 0.586610354026619 | 1.0005(0.9986-1.0024) |
|  | FOXO3 | MR Egger | 14 | 0.236243475008589 | 1.0012（0.9993-1.0032） |
|  |  | Weighted median | 14 | 0.615499745283389 | 1.0002（0.9993-1.0011） |
|  |  | Inverse variance weighted | 14 | 0.819694556797455 | 0.9999（0.9993-1.0006） |
|  |  | Simple mode | 14 | 0.272511725136149 | 0.9991（0.9976-1.0006） |
|  |  | Weighted mode | 14 | 0.608296047957791 | 1.0002（0.9994-1.0011） |

Supplementary Table 9. Primer sequences for qPCR

| Genus | Primers |  | Sequence (5’-3’) |
| --- | --- | --- | --- |
| mouse | *Actb* | Forward | CTACCTCATGAAGATCCTGACC |
|  |  | Reverse | CACAGCTTCTCTTTGATGTCAC |
| mouse | *Tnf* | Forward | ATGTCTCAGCCTCTTCTCATTC |
|  |  | Reverse | GCTTGTCACTCGAATTTTGAGA |
| mouse | *Il1* | Forward | CACTACAGGCTCCGAGATGAACAAC |
|  |  | Reverse | TGTCGTTGCTTGGTTCTCCTTGTAC |
| mouse | *Il6* | Forward | CTCCCAACAGACCTGTCTATAC |
|  |  | Reverse | CCATTGCACAACTCTTTTCTCA |
| mouse | *Tjp1* | Forward | TGTAGACGATCATCCACCCAAAGC |
|  |  | Reverse | ATTAGGCAGAGCACCATCAGAAGG |
| mouse | *Ocln* | Forward | CCTCTGACCTTGAGTGTGGATGAC |
|  |  | Reverse | TCCTCTTGCCCTTTCCTGCTTTC |
| mouse | *Il22* | Forward | ATGAGTTTTTCCCTTATGGGGAC |
|  |  | Reverse | GCTGGAAGTTGGACACCTCAA |
| mouse | *Nmnat1* | Forward | CCCAAGAGCCCAAACCAACAG |
|  |  | Reverse | CGAGTGATACAGATGAGCCCAAAG |
| mouse | *Nmnat2* | Forward | GAGGCAGATATGGAAGTGATTGTTGG |
|  |  | Reverse | TTGCGGAGTATGGAGGAGTGATTC |
| mouse | *Nmnat3* | Forward | TTCGGCTTGGTGTGCGTGAG |
|  |  | Reverse | TGCTGAAACTGCTGGAGGATGG |
| mouse | *Il22-1* | Forward | AAGATGAAAGAGGCAGAC |
|  |  | Reverse | CTGGGAAACTAACAAACA |
| mouse | *Il22-2* | Forward | AGGCTGAGTGCTTGTATT |
|  |  | Reverse | TTCCCATGTCTGATTGTC |
| mouse | *Il22-3* | Forward | AGTGGAATGGCGAAGAGG |
|  |  | Reverse | CAGGAGCCGAAGGTGAGA |
| mouse | *Il22-4* | Forward | GTCCGCTGCTGTCCAACAGA |
|  |  | Reverse | TCTGTTGGACAGCAGCGGAC |
| mouse | *Il22-5* | Forward | TGAGGATGCTGGGAGTTA |
|  |  | Reverse | GAGGTGGCAGTGAGTGAA |
| mouse | *Il22-6* | Forward | CTGCCAAGGATACACCAA |
|  |  | Reverse | CTAGACTAGAACGCGAAG |
| mouse | *Il22-7* | Forward | TTCTCCAGTGTCCCTCTA |
|  |  | Reverse | CACGCAAATGCCTACATC |
| mouse | *Il22-8* | Forward | ATGCAATCAATCCCAGTA |
|  |  | Reverse | AAATCATAGCCAAGGTCA |
| mouse | *Il22-9* | Forward | ACTTGACCTTGGCTATGA |
|  |  | Reverse | ATGTTGGCTTTCTTCACA |
| mouse | *Il22-10* | Forward | TCTATGAAGTTGGTGGGA |
|  |  | Reverse | GCTATTGAGTCAGCGTTT |
| mouse | *Sirt1* | Forward | gagctggggtttctgtctcc |
|  |  | Reverse | aacatggcttgagggtctgg |
| mouse | *Sirt2* | Forward | GAGGTGGCATGGATTTTGAC |
|  |  | Reverse | AGATGGTAGTGCTGGGGTTG |
| mouse | *Sirt3* | Forward | tcacaaccccaagccctttt |
|  |  | Reverse | tatagagccgcagaagcagc |
| mouse | *Sirt4* | Forward | GGCCGGACCAGAAGCCTTT |
|  |  | Reverse | TTGCAGCCTTCACAGGTCAT |
| mouse | *Sirt5* | Forward | CTCCGGGCCGATTCATTTCC |
|  |  | Reverse | GCGTTCGCAAAACACTTCCG |
| mouse | *Sirt6* | Forward | gacctgatgctcgctgatga |
|  |  | Reverse | ctggcggtcatgttttgtgg |
| mousse | *Sirt7* | Forward | CCTGCATCCCTAACAGAGAG |
|  |  | Reverse | AGCTGGACCCTAAACACAGG |
| mouse | *Csf2* | Forward | CCGCTCACCCATCACTGTCAC |
|  |  | Reverse | GGCATGTCATCCAGGAGGTTCAG |
| Human | *ACTB* | Forward | CAGGCAGCTCGTAGCTCTT |
|  |  | Reverse | GGAAATCGTGCGTGACATT |
| Human | *TJP1* | Forward | GCGGATGGTGCTACAAGTGATG |
|  |  | Reverse | GCCTTCTGTGTCTGTGTCTTCATAG |
| Human | *OCLN* | Forward | CCTCTGACCTTGAGTGTGGATGAC |
|  |  | Reverse | TCCTCTTGCCCTTTCCTGCTTTC |
