# Supplementary material for: Rhoifolin Alleviates Ulcerative Colitis by Targeting NMNAT1 and Activating SIRT1‐FOXO1 Signaling to Enhance ILC3s Effector Function
Source: Adv Sci (Weinh). 2026 Aug 7;13(60):e76935. doi: 10.1002/advs.76935 (PMC13449538; doi:10.1002/advs.76935)
Supplement: Supplementary file 2 [file ADVS-13-e76935-s002.docx]

**Rhoifolin Alleviates Ulcerative Colitis by Targeting NMNAT1 and Activating SIRT1-FOXO1 Signaling to Enhance ILC3s Effector Function**

**Running title: Rhoifolin boosts ILC3s response through targeting NMNAT1**

Hongqiong Yang**^†,^**^a^, Yishu Zhang**^†,^**^a^, Sijia Feng**^†,^**^a^, Xiaoqing Ma^a^, Jiayi Feng^a^, Junwei Wang^a^, Jialin Gao^a^, An Pan^a^, Dapeng Wu^b^, Kang Ding^c^, Caijuan Zheng^*,d^, Qi Lv^*,a^, Lihong Hu^*,a^

*^a^* Jiangsu Key Laboratory for Functional Substance of Chinese Medicine, School of Pharmacy, Nanjing University of Chinese Medicine, Nanjing, 210023, P. R. China

*^b^* Jiangsu Province Hospital of Chinese Medicine, Affiliated Hospital of Nanjing University of Chinese Medicine, Nanjing, 210029, P.R. China

*^c^* Nanjing Hospital of Chinese Medicine Affiliated to Nanjing University of Chinese Medicine, Nanjing, 210000, P. R. China.

*^d^* Key Laboratory of Tropical Medicinal Plant Chemistry of Ministry of Education, College of Chemistry and Chemical Engineering, Hainan Normal University, Haikou, China.

**^†^** The authors have contributed equally to this work and share first authorship.

***** *Correspondence:*

*Caijuan Zheng*, *Qi Lv*, *Lihong Hu*

*Email:* [*cncaijuan2002@163.com*](mailto:dingkang@njucm.edu.cn;%20cncaijuan2002@163.com); [*qilv@njucm.edu.cn*](mailto:qilv@njucm.edu.cn)*;* *lhhu@njucm.edu.cn*

**Supplementary Materials and Methods**

**2.1 TNBS-induced colitis**

Colitis was induced in mice using 2, 4, 6-trinitrobenzenesulfonic acid (TNBS, P2297, Sigma-Aldrich, St. Louis, MO, USA) as previously described. Briefly, mice were lightly anesthetized, and TNBS solution (2.5 mg in 50 μL of 50% ethanol) was administered intrarectally using a flexible catheter inserted 4 cm from the anus. Mice were held in a vertical position for 1-2 min to ensure uniform distribution of TNBS. To investigate the protective effects of Rhoifolin, mice were randomly assigned to six groups: Normal, TNBS, Rhoifolin (1, 3, 10 mg/kg) and 5-ASA (200 mg/kg). Rhoifolin and 5-ASA were administered by oral gavage starting on the day of TNBS induction and continued for 7 consecutive days. Mice in the Normal and TNBS-groups received an equivalent volume of 0.5% carboxymethylcellulose sodium (CMC-Na) as vehicle. Clinical parameters, colon length, and histopathological scores were assessed at the end of the treatment period to evaluate the efficacy of Rhoifolin.

**2.2 Histopathological assessment**

Colonic tissues were harvested, rinsed with ice-cold PBS, and fixed in 4% paraformaldehyde at 4 °C for 24 h. The tissues were then embedded in paraffin, sectioned at 5 μm thickness, and stained with hematoxylin and eosin (H&E) following standard protocols. Histopathological evaluation was performed under a light microscope by two independent pathologists, who were blinded to the experimental groups, ensuring unbiased assessment of tissue morphology and inflammatory changes.

**2.3 Establishment of colonic epithelial cell monolayer model**

The caco-2 cells were cultured in DMEM medium supplemented with 10% fetal bovine serum (FBS), 1% penicillin-streptomycin, 1% L-glutamine, and maintained at 37 °C in a 5% CO₂ incubator. The culture medium was replaced every 2-3 days, and cells were passaged upon reaching 70-80% confluence. To establish a polarized epithelial monolayer, cells were seeded onto the apical side of a 6.5 mm trans-well insert with a 0.4 μm pore size (3470, Corning, NY, USA). For differentiation and tight junction formation, cells were cultured in serum-free medium for 21 days, with medium changes every two days. The integrity of the epithelial barrier was evaluated by measuring transepithelial electrical resistance (TEER) using an EVOM2 meter at 7, 14, and 21 days.

**2.4 Machine learning**

To identify potential SIRT1-associated targets relevant to UC, we first intersected the list of SIRT1-interacting proteins with the expressed genes derived from the GEO dataset GSE87466. Two complementary machine learning approaches were then applied to refine feature selection and highlight biologically significant candidates. First, the Random Forest (RF) algorithm was implemented to evaluate the relative contribution of each gene, generating an important ranking based on its ability to discriminate between disease and control samples. Second, a Support Vector Machine-Recursive Feature Elimination (SVM-RFE) model was constructed to iteratively remove irrelevant or redundant features, thereby optimizing the predictive subsets of genes. A five-fold cross-validation strategy was incorporated to ensure model robustness and prevent overfitting. Genes consistently recognized as top-ranked by both algorithms were defined as core hub genes, representing potential key mediators underlying the therapeutic effects of Rhoifolin against UC.

**2.5 Mendelian Randomization analysis**

Mendelian randomization (MR) was employed to explore the potential causal relationship between genetically predicted gene expression and the risk of UC. This approach utilizes genetic variants that serves as instrumental variables (IVs), minimizing confounding and reverse causation inherent to traditional observational studies. The MR analysis was conducted in R software (version 4.4.3) using the TwoSampleMR package. Summary statistics for single nucleotide polymorphisms (SNPs) associated with the candidate genes were obtained from the GWAS Catalog (https://gwas.mrcieu.ac.uk/), which provides harmonized genome-wide association datasets. The primary causal estimates were derived using the inverse variance weighted (IVW) approach, which assumes that all genetic instruments satisfy the conditions of relevance, independence, and exclusion restriction. To evaluate the consistency and reliability of causal inference, multiple sensitivity analyses were performed. The MR-Egger regression was used to test for potential directional pleiotropy, while the Leave-One-Out (LOO) analysis assessed the influence of individual SNPs on the overall causal estimate. These complementary analyses ensured the robustness and validity of the MR findings.

**2.6 Molecular docking analysis**

The X-ray crystal structure of NMNAT1 was retrieved from the Uniprot database and prepared using the Protein Preparation module in Schrodinger Maestro 2019. The polar hydrogens were added, water molecules were removed, and the protonation states of residues were optimized. The protein structure was subsequently minimized using the OPLS 2005 force field. The binding pocket was defined as all amino acid residues within 20Å of the native ligand. The ligand Rhoifolin was initially constructed by ChemBio 3D Ultra software and further processed using the LigPrep module in Schrodinger Maestro 2019. Molecular docking was performed using Glide in standard precision mode, generating up to five poses per ligand. Glide scores were used to evaluate binding affinity. The resulting docking poses and interactions, including hydrogen bonds, hydrophobic contacts, and overall binding stability, were visualized and analyzed using PyMOL.

**2.7 Molecular dynamics simulation**

Molecular dynamics (MD) simulations (500 ns) were performed using the Desmond module in Schrodinger Maestro 2019. Therhoifolin-NMNAT1 complex, obtained from the Glide docking pose, was parameterized using the OPLS3 force field. The complex was solvated in an orthorhombic box with a 10 Å buffer in all direction (x, y and z) using transferable interaction potential (TIP3P) water molecules. The system was neutralized by adding Na^+^ counterions to balance the net charge. A 500 ns of MD simulations was then carried out to assess the dynamic behavior and structural stability of the complex. Structural deviations were quantified by calculating the root-mean-square deviation (RMSD) and energy ﬂuctuation, while local flexibility was assessed *via* root-mean-square fluctuation (RMSF) of individual NMNAT1 residues. Hydrogen bonding patterns and hydrophobic interactions were analyzed using the Simulation Interactions Diagram module in Schrodinger Maestro 2019.

The binding free energies were estimated using the molecular mechanics/generalized Born surface area (MM/GBSA) module in Schrodinger Maestro 2019. The Generalized Born (GB) term was calculated with the GB continuum model, while the surface area (SA) contribution was estimated using the solvent-accessible surface area (SASA) approach. An ensemble of 1000 snapshots from the final 10 ns of the MD trajectory was used for MM/GBSA calculations, with an energy gradient convergence threshold of 0.5. A total of 100 frames were used for each normal-mode analysis.

**2.8 Cellular Thermal Shift Assay (CETSA)**

CETSA was performed to evaluate the thermal stability of NMNAT1 or NAMPT in the presence of the Rhoifolin. Briefly, cells were harvested and lysed with NP-40 lysis buffer (P0013F, Beyotime, Shanghai, China). The lysates were centrifuged at 20,000 × g for 15 min at 4 °C to remove debris. Supernatants were aliquoted and incubated with the Rhoifolin or DMSO at room temperature for 1 hour. The samples were then heated at a temperature gradient (46-64 °C, 3 min each) using a thermal cycler, followed by cooling to room temperature for 3 min. Aggregated proteins were removed by centrifugation at 20,000 × g for 20 min at 4 °C, and the soluble fractions were collected. Protein levels of NMNAT1 or NAMPT were analyzed by western blotting assay.

**2.9 Solvent-induced precipitation (SIP) assay**

Cell lysates were prepared in NP-40 lysis buffer (P0013F, Beyotime, Shanghai, China) and clarified by centrifugation at 20,000 × g for 15 min at 4 °C. Proteins were precipitated by adding an organic solvent mixture of acetone/ethanol/acetic acid (A.E.A.) with ratio of 50: 5: 0.1 to reach the final percentage of organic solvent ranging from 4%-12%. Subsequently, the mixtures were equilibrated at 800 rpm (ThermoMixer C, Thermo Fisher, Waltham, MA, USA) for 20 min at 37 °C. Aggregated proteins were removed by centrifugation at 20,000 × g for 20 min at 4 °C, and the soluble fractions were collected. Protein levels of NMNAT1 or NAMPT were analyzed by western blotting assay.

**2.10 NMNAT1 enzymatic activity assay**

NMNAT1 enzymatic activity was measured using the NMNAT1 Activity Assay Kit (Colorimetric, ab221820, Abcam, Cambridge, UK) according to the manufacturer’s instructions. Briefly, the purified NMNAT1 protein (50 µg) was incubated with the provided reaction mixture containing all four enzymes and substrates in a 96-well plate at 37 ℃. The formation of WST-1 formazan was measured at OD450 nm at regular intervals to determine the reaction velocity.

**2.11 SIRT1 enzymatic activity assay**

SIRT1 enzymatic activity was measured using the SIRT1 Activity Assay Kit (Fluorometric, ab156065, Abcam, Cambridge, UK) according to the manufacturer’s instructions. The cells were harvested and lysed in the provided lysis buffer. Protein concentrations were determined by using the BCA assay, and equal amounts of lysate were incubated with the acetylated substrate peptide and NAD+ at 37 ℃ for 30-60 min. the deacetylation reaction generates a fluorescent signal that was measured using a microplate reader (excitation/emission: 360/460 nm)

**2.12 qPCR analysis**

Total RNA was extracted from colons or ILC3 using Trizol (R411-01, Vazyme, Nanjing, China) following the manufacturer’s protocol. RNA purity and concentration were assessed using a NanoDrop 2000 (Thermo Fisher Scientific, Waltham, MA, USA). Equal amounts of RNA (1 µg) were reverse transcribed into cDNA using the HiScript III RT SuperMix for qPCR (R323-01, Vazyme, Nanjing, China) under optimized reaction conditions. qPCR was performed using Hieff® qPCR SYBR Green Master Mix (11201ES08, Yeasen, Shanghai, China) in LightCycler^®^96 (Roche, Basel, Switzerland). Each reaction (final volume: 20 µL) contained 10 µL of 2× SYBR Green Mix, 0.4 µM of each forward and reverse primer, and 2 µL of diluted cDNA template. The cycling conditions were set as follows: initial denaturation at 95 °C for 5 min, followed by 40 cycles of 95 °C for 15 s and 60 °C for 30 s. Relative expression levels of target genes were calculated using the 2^^(-ΔΔCt^) method, with *Actb* serving as the internal control. The primers in the present study were listed in Supplementary Table 7.

**2.13 Western blotting**

Proteins were extracted from colons using NP-40 lysis buffer (P0013F, Beyotime, Shanghai, China) supplemented with protease and phosphatase inhibitors. Samples were incubated on ice for 30 min and centrifuged at 12,000 × g for 15 min at 4 °C to collect the supernatant. Protein concentration was determined using the enhanced BCA protein assay kit (P0010S, Beyotime, Shanghai, China) following the manufacturer’s instructions. The proteins were mixed with 5× loading buffer, boiled at 100 °C for 5 min, and separated by SDS-PAGE on a 10% polyacrylamide gel. Proteins were then transferred onto a PVDF membrane using a wet transfer system. Membranes were blocked in 5% non-fat milk in TBST at room temperature for 2 h, followed by overnight incubation at 4 °C with primary antibodies targeting SIRT1 (ab189494, Abcam, Cambridge, UK), FOXO1 (2880, CST, Danvers, MA, USA), NMNAT1 (YN6677, ImmunoWay, TX, USA), NAMPT (YT6086, ImmunoWay, TX, USA) at appropriate dilutions of 1:1000. After washing three times with TBST, membranes were incubated with horseradish peroxidase (HRP)-conjugated secondary antibodies (7076, anti-mouse, 7074, anti-rabbit, CST, Danvers, MA, USA) at room temperature for 1 h. Protein bands were visualized using an enhanced chemiluminescence (ECL) detection kit (36222ES, Yeasen, Shanghai, China) and imaged with a Tanon 5200 Multi (Tanon, Shanghai, China). Densitometric analysis was performed using ImageJ (NIH, Bethesda, MD, USA), and target protein expression was normalized to β-actin.

**2.14 Immunofluorescence staining**

Freshly collected colonic sections and cells were fixed with 4% paraformaldehyde for 15 min at room temperature, permeabilized with 0.1% Triton X-100 (P0096, Beyotime, Shanghai, China) in PBS for 20 min, and blocked with 3% BSA for 1 h. Primary antibodies including ZO-1 (13663, CST, Danvers, MA, USA), Occludin (91131, CST, Danvers, MA, USA), Claudin-1 (13995, CST, Danvers, MA, USA) were added at the dilution of 1:100 and incubated at 4 °C overnight. Coverslips were washed and incubated with Alexa Fluor 488 labeled secondary antibodies (704060, Thermo Fisher Scientific, Waltham, MA, USA) for 1 h in the dark. After DAPI (G1407, Servicebio, Wuhan, China) staining at room temperature for 8 min, the fluorescence signals were visualized with a TCS SP8 X (Leica, Wetzlar, Germany).

**2.15 CCK8 and MTT assays**

Cell viability was evaluated using the Cell Counting Kit-8 (CCK-8) and MTT assays according to the manufacturer’s instructions. Briefly, the cells were seeded into 96-well plates at a density of 5 × 10^6^ cells/mL in 100 µL of complete medium per well and allowed to adhere overnight. After treatment with Rhoifolin (1, 3, 10, 30 μM) for 48 h, 10 µL of CCK-8 reagent was added to each well, and the plates were incubated at 37 °C for 2 h. The absorbance at 450 nm was measured using a microplate reader (Thermo Fisher Scientific, Waltham, MA, USA). On the other hand, 20 µL of 5 mg/mL MTT reagent (475989, Sigma-Aldrich, St. Louis, MO, USA) was added to each well, and the plates were incubated at 37 °C for 4 h. The supernatant was then carefully removed, and 150 µL of dimethyl sulfoxide (DMSO) was added to dissolve the formazan crystals. The absorbance at 570 nm was measured using a microplate reader. The cell viability was calculated as a percentage relative to the untreated control group.

**Supplementary Figures**

**Supplementary Figure 1. Rhoifolin significantly alleviates TNBS-induced colitis.** Acute colitis was induced in male BALB/c mice by intrarectal administration of 2.5 mg TNBS dissolved in 50% ethanol. Rhoifolin (1, 3, 10 mg/kg) was administered once daily by oral gavage for 7 consecutive days. (A) Colon length. (B) MPO activity. (C) Representative H&E-stained colon sections and histological scores. (D) Macroscopic scores. (E) Flow cytometric analysis of neutrophil infiltration in colonic lamina propria. Data were presented as means ± S.E.M of six mice in each group. ^##^*P* < 0.01 *vs.* Normal group; ^*^*P* < 0.05, ^**^*P* < 0.01 *vs.* TNBS group.

**Supplementary Figure 2. Rhoifolin ameliorates DSS-induced chronic colitis.** Chronic colitis was induced by three repeated cycles of 2% DSS exposure for 5 days followed by regular drinking water for 10 days. Rhoifolin was administered at the indicated doses throughout the entire experimental period. (A) Body weight change. (B) Colon length. (C) MPO activity in colons. (D) Representative H&E-stained colon sections and histological scores. (E) Representative flow cytometry plots and quantification of CD11b⁺Ly6G⁺ neutrophils in colonic lamina propria. (F) Representative colonoscopic images showing mucosal inflammation and injury. (G) Relative mRNA expression of pro-inflammatory cytokines, including *Tnf*, *Il1*, and *Il6*, in colons. Data were presented as means ± SEM of six mice in each group (n=6). *^##^P <* 0.01 *vs.* Normal group; *P <* 0.05, *P <* 0.01 *vs.* DSS group.

**Supplementary Figure 3. Oral administration of Rhoifolin is well tolerated in mice.** The healthy female C57BL/6 mice were orally administered Rhoifolin (1, 3, 10 mg/kg) daily for consecutive 30 days. (A) Body weight change. (B) Colon length. (C, D) Levels of aspartate aminotransferase (AST), alanine aminotransferase (ALT), urea and creatinine in serum. (E) Histopathological examination of heart, liver, spleen, lung, kidney and colon. (F-K) Flow cytometric analysis of splenic immune cell populations, including CD3^+^ CD4^+^ T cells, CD3^+^ CD8^+^ T cells, CD19^+^ B cells, CD11b^+^ Ly6C^+^ monocytes, CD11b^+^ Ly6G^+^ neutrophils and CD11b^+^F4/80^+^ macrophages. Data were presented as means ± S.E.M of six mice in each group (n=6).

**Supplementary Figure 4. Rhoifolin does not directly restore epithelial barrier integrity *in vitro*.** (A-C) Caco-2 cells were treated with TNF-α (20 ng/mL) and Rhoifolin (1, 3, 10, 30 μM) for 24 h. Representative transmission electron microscopy images (A); representative immunofluorescence images of ZO‐1 and Occludin (B, C). (D) Schematic representation of colonic organoid culture system. (E-G) Colonic organoids were treated with DSS and Rhoifolin (1, 3, 10, 30 μM) for 24 h. The morphological change of colonic organoids under DSS stimulation (E); representative immunofluorescence images of ZO-1 and Occludin in colonic organoids (F, G). Data were presented as means ± S.E.M. from three independent *in vitro* experiments (n=3).

**Supplementary Figure 5. Rhoifolin does not alter intestinal ILCs composition, ILC3s subset distribution, ILC3s survival, proliferation, recruitment, or upstream progenitor output.** (A, B) C57BL/6 mice were subjected to DSS-induced acute colitis, and Rhoifolin was administered once daily by oral gavage for 10 consecutive days. Flow cytometric analysis of the proportions of ILC1s and ILC2s in colonic lamina propria (A); flow cytometric analysis of major colonic ILC3 subsets, including CCR6⁺NKp46^-^ LTi-like ILC3s, CCR6^-^NKp46⁺ NCR⁺ ILC3s, and CCR6^-^NKp46^-^ double-negative ILC3s (B). (C-E) ILC3s was treated with Rhoifolin for 48 h, cell viability, proliferation and apoptosis were detected by MTT, CCK8 and Annexin-V/PI, respectively. (F) ILC3s were isolated from spleens of donor mice and labeled with fluorescent dye Dil. The labeled ILC3s were then intravenously adoptively transferred into colitis recipient mice. Five days post-transfer, mice were euthanized, and colons were harvested. The distribution of transferred ILC3s within the colons was visualized using immunofluorescence microscopy. (G, H) ILC3s were isolated from spleens and then treated with Rhoifolin for 48 h, the protein expression of CCR9 and CCR6 was detected by flow cytometry assay. (I) C57BL/6 mice were subjected to DSS-induced acute colitis, and Rhoifolin was administered once daily by oral gavage for 10 consecutive days. The bone marrow-derived single-cell suspensions were prepared, and frequencies of αLPs, defined as live Lin^-^CD127⁺c-kit⁺ cells, and ILCPs were determined by flow cytometry assay. Data were presented as means ± S.E.M from three independent *in vitro* experiments and six mice per group *in vivo* experiments (n=6 *in vivo* and n=3 *in vitro* experiments). *^##^P <* 0.01 *vs.* Normal group.

**Supplementary Figure 6. NMNAT1 is the predominant NMNAT isoform mediating the effect of Rhoifolin in ILC3s.** (A) The mRNA expression of *Nmnat1*, *Nmnat2*, and *Nmnat3* in ILC3s was determined by qPCR assay. (B-D) The ILC3s were transfected with siRNAs targeting *Nmnat1*, *Nmnat2*, or *Nmnat3* and then treated with Rhoifolin (30 μM) for 48 h. The mRNA expression of *Il22* was analyzed by qPCR assay. Data were presented as means ± S.E.M. from three independent experiments (n = 3). ^**^*P* < 0.01 *vs.* Normal group; ^$$^*P* < 0.01 *vs.* Rhoifolin group; ns. No significance.

**Supplementary Figure 7. FOXO1 was the potential interaction protein responsible for Rhoifolin-enhanced ILC3s effector function.** (A-D) The random forest (RF) and support vector machine (SVM) models were employed to prioritize the functional relevance of the ten predicated SIRT1-interacting proteins. (E) Venn diagram showing the overlap proteins identified by both RF and SVM models. (F-I) Mendelian randomization analysis was performed to evaluate potential causal roles of candidate gene FOXO1 in UC progression.

**Supplementary Figure 8. Mendelian randomization analysis evaluating the causal association between ATG7, FOXO3, PPARG and ulcerative colitis (UC).** (A-L) MR analysis of genetically predicted ATG7, FOXO3, PPARG expression and UC risk. Effect estimates were derived using inverse variance-weighted (IVW) models, with MR-Egger and weighted median methods included as sensitivity analyses.
